# Unveiling Gut Homeostasis Disruption in Sepsis: Towards an Integrated Mechanistic and Translational Roadmap

**DOI:** 10.1111/cpr.70253

**Published:** 2026-06-28

**Authors:** Yichen Bao, Lin Qi, Guijun Zou, Xingpeng Yang, Yizhao Ma, Zhifu Li, Xiaohui Du, Pengyue Zhao

**Affiliations:** ^1^ Department of General Surgery First Medical Center of the Chinese PLA General Hospital Beijing China; ^2^ School of Medicine Nankai University Tianjin China

**Keywords:** bacterial translocation, dysregulation of immunity, gut‐target axis, intestinal barrier, sepsis, sepsis‐induced intestinal injury

## Abstract

Sepsis, a life‐threatening clinical syndrome precipitated by a maladaptive host response to infection, is associated with substantial morbidity and high mortality rates. Gastrointestinal injury has gained recognition as a pivotal factor driving sepsis progression. Pathogen invasion incites oxidative stress and inflammatory cascades, leading to compromised intestinal barrier integrity and dysregulated local immunity. This results in increased gut permeability and bacterial translocation, fostering a state that can be described as ‘enteric sepsis’. Moreover, multi‐organ crosstalk via the gut‐liver and gut‐brain axes substantially amplifies systemic inflammation. The pathophysiology of sepsis‐induced intestinal injury is not yet fully elucidated, and clinically applicable biomarkers or early diagnostic tools remain scarce. Targeted therapeutic strategies have yet to be validated in clinical practice. This article presents a comprehensive review of recent advances in the pathophysiology and underlying mechanisms of sepsis‐induced intestinal injury, focusing on the signalling networks that disrupt intestinal homeostasis and immune equilibrium. Particular emphasis is placed on identifying key pathways and candidate biomarkers for early diagnosis and intervention. Additionally, the therapeutic potential of targeted intestinal‐protective agents is evaluated, integrating insights from traditional Chinese medicine to propose a combined treatment strategy. Ultimately, this review aims to establish a translational framework to advance clinical management and therapeutic innovation for sepsis‐associated intestinal dysfunction.

## Graphical abstract

Elucidating the contribution of gut‐organ axes will provide new insights for developing combined therapeutic strategies against sepsis‐associated multiple organ dysfunction.

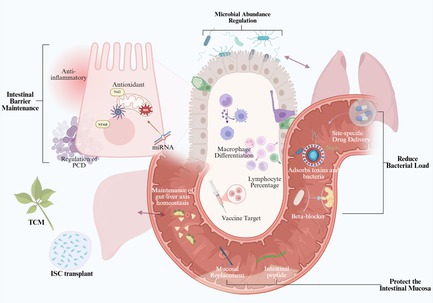

**Abbreviations**
AGI: acute gastrointestinal injury
AhR: aryl hydrocarbon receptor
AKT: AKT serine/threonine kinase (also known as protein kinase B)
ALI: acute lung injury
AMP: antimicrobial peptide
AMPK: AMP‐activated protein kinase
AP‐1: activator protein 1
AQP: aquaporin
ARE: antioxidant response element
ASC: apoptosis‐associated speck‐like protein containing a CARD
ASPEN: American Society for Parenteral and Enteral Nutrition
ATF: activating transcription factor
BBR: berberine
BMP: bone morphogenetic protein
BNP: B‐type natriuretic peptide
CAT: catalase
CCL: C‐C motif chemokine ligand
CEO: chimonanthus nitens oliv essential oil
cGAMP: cyclic GMP‐AMP
cGAS: cyclic GMP‐AMP synthase
CHIP: C‐terminus of HSC70‐interacting protein
CHOP: C/EBP homologous protein (gene name DDIT3)
CNS: central nervous system
CRB3: Crumbs3
CRP: C‐reactive protein
CTLA‐4: cytotoxic T‐lymphocyte‐associated protein 4
CX3C: C‐X3‐C motif chemokine
CX3CL1: C‐X3‐C motif chemokine ligand 1 (fractalkine)
CX3CR1: C‐X3‐C motif chemokine receptor 1
DAMP: damage‐associated molecular pattern
DAO: diamine oxidase
DIZE: diminazene aceturate
DSS: dextran sulphate sodium
ECM: extracellular matrix
EEC: enteroendocrine cell
EFHD2: EF‐hand domain family member D2 (swiprosin‐1)
ENS: enteric nervous system
ER: endoplasmic reticulum
ERK: extracellular signal‐regulated kinase
ESPEN: European Society for Clinical Nutrition and Metabolism
FAK: focal adhesion kinase
Family 1: CYP1A1: cytochrome P450
FasL: fas ligand
FGF: fibroblast growth factor
FMT: faecal microbiota transplantation
FoxP3: forkhead box P3
FPR1: formyl peptide receptor 1
FXR: farnesoid X receptor
GALT: gut‐associated lymphoid tissue
GGA: geranylacetone
GLA: γ‐linolenic acid
GLP‐1: glucagon‐like peptide‐1
GM‐CSF: granulocyte‐macrophage colony‐stimulating factor
GPX: glutathione peroxidase
GSDMD: gasdermin D
GSH: glutathione
GSNO: S‐nitrosoglutathione
HIF‐1α: hypoxia‐inducible factor 1‐alpha
HMGB1: high mobility group box 1
HO‐1: heme oxygenase‐1
HPA: hypothalamic–pituitary–adrenal
HSP: heat shock protein
I/R: ischemia–reperfusion
IAP: intestinal alkaline phosphatase
ICU: intensive care unit
IEC: intestinal epithelial cell
I‐FABP: intestinal fatty acid‐binding protein
IFN: interferon
IFNAR1: interferon alpha and beta receptor subunit 1
IgM: immunoglobulin M
IKK: IκB kinase
IL: interleukin
iNOS: inducible nitric oxide synthase
IPA: indole‐3‐propionic acid
IRF: interferon regulatory factor
ISC: intestinal stem cell
IκB: inhibitor of kappa B
JAK: Janus kinase
JNK: c‐jun N‐terminal Kinase
Keap1: Kelch‐like ECH‐associated protein 1
LA: linoleic acid
LPS: lipopolysaccharide
MAM: mitochondria‐associated endoplasmic reticulum membrane
MAPK: mitogen‐activated protein kinase
MDA: malondialdehyde
MDSC: myeloid‐derived suppressor cell
MFG‐E8: milk fat globule‐EGF Factor 8 (lactadherin)
MFN2: mitofusin 2
miRNA: microRNA
MLC: myosin light chain
MLCK: myosin light chain kinase
MLKL: mixed lineage kinase domain‐like protein
MLN: mesenteric lymph node
MMV: membrane‐derived microvesicle
MODS: multiple organ dysfunction syndrome
MOMP: mitochondrial outer membrane permeability
MPTP: mitochondrial permeability transition pore
MUC: mucin
MyD88: myeloid differentiation primary response 88
NaB: sodium butyrate
NET: neutrophil extracellular trap
NF‐κB: nuclear factor kappa‐light‐chain‐enhancer of activated B cells
NHE3: sodium‐hydrogen exchanger 3
NHH: non‐hepatic hyperammonemia
NLR: NOD‐like receptor
NLRP3: NOD‐, LRR‐ and pyrin domain‐containing protein 3
NOX: NADPH oxidase
Nrf2: nuclear factor erythroid 2‐related factor 2
NUFIP1: nuclear FMR1 interacting protein 1
OCA: obeticholic acid
PAMP: pathogen‐associated molecular pattern
PANoptosis: pyroptosis, apoptosis and necroptosis
PCD: programmed cell death
PCT: procalcitonin
PD‐1: programmed cell death protein 1
PGC‐1α: peroxisome proliferator‐activated receptor gamma coactivator 1‐alpha
PI3K: phosphatidylinositol 3‐kinase
PINK1: PTEN‐induced putative kinase 1
PLT: platelet
Polypeptide 1: subfamily A
PPA: phenylpyruvic acid
PPARγ: peroxisome proliferator‐activated receptor gamma
PROS1: protein S
rhBNP: recombinant human B‐type natriuretic peptide
RIPK: receptor‐interacting protein kinase
ROCK: rho‐associated coiled‐coil containing protein kinase
ROS: reactive oxygen species
SAE: sepsis‐associated encephalopathy
SCFA: short‐chain fatty acid
SDH: succinate dehydrogenase
sIgA: secretory immunoglobulin A
SIRT2: sirtuin 2
SOCS: suppressor of cytokine signalling
SOD: superoxide dismutase
STAT: signal transducer and activator of transcription
STING: stimulator of interferon genes
TA: transit‐amplifying (cell)
TAM: tight junction‐associated MARVEL protein
TAMP: tight junction‐associated MARVEL protein
T‐AOC: total antioxidant capacity
TCA: tricarboxylic acid cycle (krebs cycle)
TCM: traditional Chinese medicine
TER: transepithelial electrical resistance
TFF: trefoil factor
Tfh: T follicular helper (cell)
TGF‐β: transforming growth factor‐beta
Th: T helper (cell)
TIM‐3: T‐cell Immunoglobulin and mucin domain‐containing‐3
TIMP‐2: tissue inhibitor of metalloproteinase 2
TIRAP: toll‐interleukin 1 receptor (TIR) domain‐containing adaptor protein
TJ: tight junction
TLR: toll‐like receptor
TNF: tumour necrosis factor
TNFR1: tumour necrosis factor receptor 1
Treg: regulatory T (cell)
TXNIP: thioredoxin‐interacting protein
UCP2: uncoupling protein 2
ULK1: Unc‐51 like autophagy activating kinase 1
VEGF: vascular endothelial growth factor
VIP: vasoactive intestinal peptide
XBP‐1: X‐box binding protein 1
ZBP‐1: Z‐DNA binding protein 1
ZO‐1: zonula occludens‐1

## 1 Introduction

The Sepsis‐3 criteria conceptualize sepsis as life‐threatening organ dysfunction stemming from a dysregulated host response to infection [1]. Recent epidemiological data (The Lancet, 2025) estimate that sepsis contributed to approximately 31.5% of global all‐cause mortality by 2021, with fatalities from underlying infectious etiologies increasing by 86.4% between 2019 and 2021 [2]. Sepsis‐related shock accounts for 10%–30% of intensive care unit (ICU) admissions and carries a mortality rate of 35%–45% [3]. Given its high incidence, substantial mortality, and significant healthcare burden, sepsis remains a pressing public health issue [4]. Consequently, the early detection of patients with immune dysregulation is essential for prompt intervention and improved clinical outcomes [5].

The intestine, as a hollow organ under continuous environmental exposure, maintains a sophisticated, multi‐layered epithelial barrier system. This defence structure comprises—from the luminal to the basolateral side—a mucus layer, antimicrobial peptides, secretory immunoglobulin A (SIgA), a monolayer of intestinal epithelial cells, regenerative crypt‐based stem cells, and resident immune cells populating the epithelial layer and underlying lamina propria [6]. Functionally, the intestinal barrier acts in concert to preserve epithelial integrity, regulate selective permeability, and sustain luminal homeostasis—including electrolyte balance, pH stability, and antimicrobial defence [7, 8]. The intestinal commensal microbiota is essential for preserving gut homeostasis, thereby bolstering host defence via the competitive exclusion of invasive pathogens, thereby limiting systemic translocation of luminal antigens, microorganisms and toxins. Immune tolerance in the gut is shaped by resident immune cells, as well as persistent exposure to commensal microbes and dietary antigens. Key regulatory mechanisms—including macrophage polarization, leukocyte trafficking, T cell differentiation, and secretory IgA production—collectively uphold humoral and cellular immune balance in the intestine [9, 10, 11], and elevate the threshold for aberrant immune activation [12]. Furthermore, exogenous factors including bile acids and nutrients also regulate intestinal processes including homeostasis and motility [13].

Under physiological conditions, the intestinal epithelium simultaneously fulfils dual roles: facilitating nutrient digestion and absorption via enzymatic cooperation with organs such as the pancreas, while maintaining a selective barrier against luminal pathogens. This functional duality is sustained by the self‐renewal property of intestinal stem cells (ISCs), which confer reparative potential after injury [14, 15]. Residing within the Lieberkühn crypt niche, ISCs respond to Wnt‐mediated signals by proliferating and differentiating into six major epithelial lineages—enterocytes, goblet cells, Paneth cells, and so on [16]. The coordination of cell proliferation, differentiation and migration along the crypt–villus axis ensures continuous epithelial renewal [8, 17]. Disruption of this process, marked by excessive epithelial apoptosis and suppressed ISCs' activity, is a hallmark of chronic gastrointestinal inflammation and sepsis‐related gut injury [18].

In sepsis induced by viral, bacterial or severe systemic insults, dysregulated host immunity can initiate a deleterious systemic inflammatory cascade, resulting in acute multi‐organ injury and dysfunction [19]. The gastrointestinal tract, as a primary target, is often characterized as a ‘motor’ or ‘driver’ of sepsis progression [9, 20]. Intestinal injury occurs in nearly half of intensive care‐treated sepsis cases and is strongly linked with septic shock and 28‐day mortality [21, 22]. Under pathogenic assault, the intestinal epithelium undergoes marked oxidative stress and immune hyperactivation, leading to innate immune dysregulation and the characteristic pathophysiological changes: (1) endothelial dysfunction; (2) establishment of a pro‐inflammatory signalling niche; (3) increased intestinal permeability due to loss of barrier integrity; (4) disruption of intestinal immune homeostasis; and (5) impaired mucosal repair. These alterations collectively facilitate bacterial translocation and perpetuated systemic inflammation, culminating in gut‐derived sepsis [13, 20].

**FIGURE 1:**
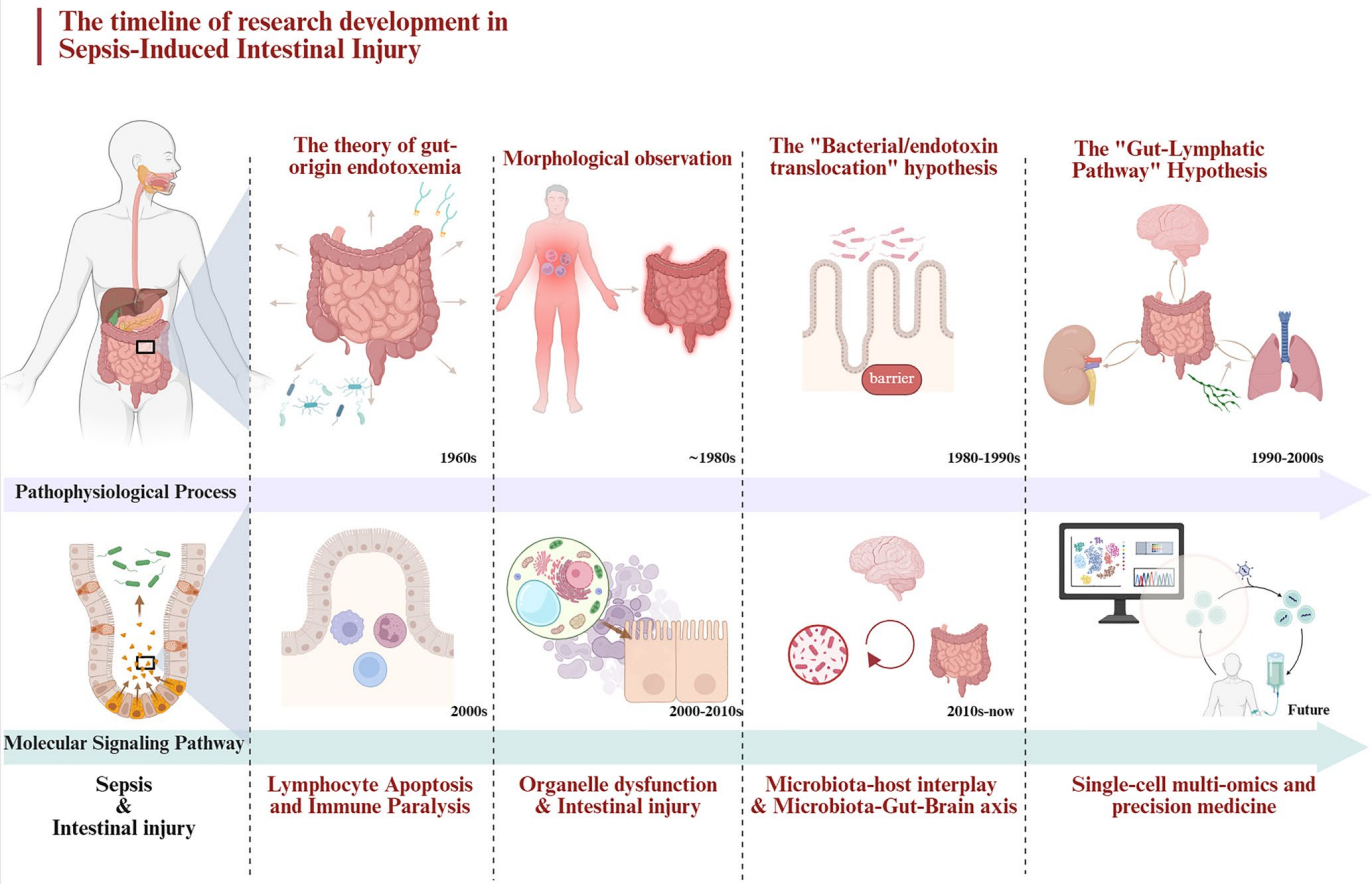
The timeline of research development in sepsis‐induced intestinal injury. Research on sepsis‐induced intestinal injury has undergone theoretical paradigm shifts and refinements, paralleling advances in biotechnology and the deepening exploration of pathophysiological mechanisms. The field has evolved from the initial ‘gut‐origin endotoxemia theory’, which first identified the intestine as a source of endotoxins, through a stage of ‘morphological observation’ that viewed the gut as a passive victim. A paradigm shift occurred with the ‘bacterial/endotoxin translocation’ hypothesis, which established the gut as the ‘motor’ driving sepsis and multiple organ dysfunction. Subsequent refinement came with the ‘gut‐lymphatic pathway’ theory, while the concept of ‘lymphocyte apoptosis and immune paralysis’ elucidated the gut's pivotal role in immunosuppression. Investigations into ‘organelle dysfunction’ further clarified precise molecular mechanisms of pathogenesis. In recent years, the research frontier has shifted towards ‘microbiota‐host interactions’ and the ‘microbiota‐gut‐brain axis’, integrating the microbiome and neural regulation into the research network. Looking forward, ‘single‐cell multi‐omics and precision medicine’ are propelling the field towards ultra‐high‐resolution mapping and individualized therapeutic strategies.

Multiple theoretical frameworks have been advanced to elucidate the pathogenesis of gut‐derived sepsis. Bidirectional ‘gut–target organ’ crosstalk models—namely the gut–liver, gut–brain, gut–lung, and gut–kidney axes—derived from intestinal lymphatic and venous drainage anatomy, explain multiple organ damage syndrome (MODS) pathogenesis in gut‐derived sepsis [23]. The ‘bacterial translocation’ hypothesis attributes the initiation of sepsis to a compromised intestinal barrier [24]. In parallel, the ‘gut‐lymphatic’ theory offers an anatomical basis for the observed similarity between respiratory and gut microbiota in patients with acute respiratory distress syndrome (ARDS), supporting the gut–lung axis theory [25, 26, 27]. More comprehensively, the ‘gut dialogue’ theory integrates injury responses involving intestinal epithelial cells (IECs), the mucosal immune system, commensal microbiota, and enteric neural circuits, offering a holistic perspective on multi‐component gut‐derived sepsis pathways [20, 28]. These theories have been partially validated in various animal models and clinical studies but present certain inconsistencies that require further investigation [29]. Figure 1 illustrates the chronological progression of research pertaining to sepsis‐induced intestinal injury.

Antibiotics and anti‐inflammatory agents remain cornerstone therapies for sepsis [30, 31]. Approximately 75% of ICU patients receive antibiotics daily. Despite serving as an indispensable double‐edged sword in anti‐infective therapy, unnecessary antibiotic administration is a key driver of gut dysbiosis and a significant contributor to mortality [32, 33]. Stepped antibiotic therapy represents a key treatment principle for sepsis and septic shock [34]. Evidence‐based guidelines from European and American society for clinical nutrition and metabolism (ESPEN and ASPEN) emphasize early enteral nutrition combined with gastrointestinal function restoration to reduce infection rates. Adjunctive probiotics further modulate gut microbiota, a strategy increasingly integrated into clinical practice [35, 36]. Despite these advancements, intestinal protection in sepsis remains challenging. Targeting the immune‐inflammation‐microbiota axis represents a promising therapeutic strategy [37], yet standardized diagnostic criteria and specific biomarkers for intestinal injury are lacking. Moreover, no targeted therapies currently exist to effectively mitigate intestinal damage in sepsis.

This review elucidates the pathophysiology of sepsis‐induced intestinal injury by delineating key molecular pathways and intercellular communication. Mechanisms governing barrier integrity, immune homeostasis, and microenvironmental stability are examined to identify high‐specificity biomarkers and therapeutic targets. By integrating emerging pharmacological strategies—including both western and traditional Chinese medicine (TCM)—this work paves the way for novel therapeutic strategies designed to maintain intestinal barrier function and enhance survival in sepsis.

## 2 Pathophysiological Features of Sepsis‐Induced Intestinal Injury

### 2.1 Ischemia–Reperfusion Injury (I/R)

The pathophysiological mechanisms of intestinal I/R injury in sepsis can be delineated into the ischemic and reperfusion phases [38]. In severe infections, systemic hemodynamic instability, markedly increased metabolic demand and oxygen consumption, and preferential perfusion of essential organs (e.g., the brain, heart and lungs) reduce intestinal perfusion pressure. This results in intestinal mucosal ischemia and hypoxia, which can rapidly damage metabolically active tissues. Endothelial cell (EC) injury, leukocyte plugging, and microthrombus formation contribute to microcirculatory dysfunction [39]. Research on the ischemic phase remains relatively limited; however, emerging evidence indicates that metabolic alterations in IECs—such as succinate accumulation—along with the activation of regulated cell death pathways, may mediate early intestinal injury [38].

Following fluid resuscitation and the restoration of systemic blood pressure, blood flow is reestablished to the ischemic intestine. However, this reperfusion process can trigger a cascade of events causing ‘secondary injury’, resulting in significant intestinal and systemic damage. Reperfusion injury is also considered a critical factor in bacterial translocation resulting from intestinal injury, which can subsequently cause remote organ damage [40]. The precise mechanisms of reperfusion injury remain incompletely understood but are thought to involve oxidative stress, inflammatory activation, bacterial translocation, regulated EC death and calcium overload [40].

These events induce endothelial dysfunction, neutrophil activation, microbial translocation, and inflammatory cascade. Neutrophil‐endothelial interactions and the vagus–sympathetic pathway may mediate gut‐derived I/R‐ALI [41, 42], and may even lead to MODS and systemic inflammatory response syndrome (SIRS) [43, 44]. The immune microenvironment and gut microbiota are critical determinants of intestinal I/R injury. L‐malic acid (MA) drives macrophage M2 polarization and restores interleukin‐10 levels in a suppressor of cytokine signalling 2 (SOCS2)‐dependent manner, thereby enhancing the success rate of intestinal organoid transplantation in repairing mucosal injury [45]. Lactobacilli stimulate interleukin‐10 (IL‐10) production, a response triggered by Toll‐like receptor 2 (TLR2) signalling, thereby alleviating ischemia–reperfusion injury [46]. Intestinal fungi ameliorate intestinal I/R injury, a mechanism involving the suppression of epithelial pyroptosis through the modulation of macrophage SAA1 expression [47]. Furthermore, accumulating evidence indicates that microbial metabolites contribute to intestinal I/R injury by driving the aberrant activation of pathways linked to oxidative stress, apoptosis, and ferroptosis [48, 49, 50, 51, 52].

### 2.2 Intestinal Endothelial Dysfunction

Programmed cell death (PCD) is a regulated cell death process executed by dedicated molecular machinery [53]. IECs utilize PCD, in conjunction with apical villus shedding, to regulate epithelial cell numbers. IECs concurrently exhibit rapid proliferation and high rates of cell death, collectively maintaining barrier integrity [54]. Aberrant IEC death is a critical driver of extensive epithelial denudation and represents a pathognomonic feature of intestinal pathologies, including inflammatory bowel disease and infectious colitis [18].

Under septic conditions, IEC injury and microcirculatory dysfunction contribute the critical pathological foundation for intestinal mucosal barrier impairment and altered permeability. This is primarily manifested by aberrant activation of PCD and excessive autophagy in IECs [55]. Oxidative stress serves as a prerequisite trigger for dysregulated induction of PCD [56]. Triggered by exogenous damage‐associated molecular patterns (PAMPs) such as endotoxins from pathogenic microbes and endogenous damage‐associated molecular patterns (DAMPs) like oxidative stress products and inflammatory cytokines, IECs can pathologically activate various PCD pathways. These encompass apoptosis, necroptosis, pyroptosis and ferroptosis, many of which are intrinsically linked to inflammatory signalling cascades [57].

The shedding of senescent IECs from the villus tip, or of stressed IECs from the basement membrane, initiates caspase‐mediated apoptotic execution. Both the epithelial luminal surface and the crypt regions exhibit high frequencies of apoptosis [54]. Owing to the continuous turnover of the intestinal epithelium, anoikis constitutes a specific form of PCD in IECs. This process is triggered by elevated extrusion pressure at villus tips, cellular detachment or aberrant cell‐extracellular matrix adhesion. Its core mechanism involves disruption of integrin‐mediated survival signalling, regulated by the mechanosensitive ion channel Piezo1. Piezo1 not only promotes the assembly of an actin‐myosin contractile ring during cell extrusion but also mediates calcium overload, thereby exacerbating apoptosis and inducing tight‐junction (TJ) disassembly [54, 57, 58].

IECs exhibit high sensitivity to tumour necrosis factor (TNF) [54]. During sepsis, activated macrophages and T cells upregulate TNF and Fas ligand (FasL) expression. These mediators, in concert with reactive oxygen species (ROS), trigger both the extrinsic and intrinsic apoptotic pathways, thereby compromising epithelial integrity. TNF further disrupts TJ, increases epithelial permeability, induces cytokines and chemokines and recruits immune cells, thereby participating in the regulation of inflammation. Lipopolysaccharide (LPS) challenge propagates inflammatory cytokine cascades via TNF and interferon (IFN) signalling [59] Combined with DAMPs released during oxidative stress, this signalling axis drives inflammatory cell death—primarily manifesting as necroptosis and pyroptosis [60]. These lytic cell death pathways culminate in membrane rupture and DAMP release, thereby establishing a vicious cycle of inflammation [56].

Intestinal I/R triggers mitochondrial failure, liberating cytoplasmic components such as mitochondrial DNA (mtDNA) which function as DAMPs, propagating inflammation and compromising intestinal barrier integrity. Specifically, cytosolic mtDNA can activate the stimulator of interferon genes (STING) signalling pathway, thereby inducing necroptosis. Targeted inhibition of necroptosis has been demonstrated to attenuate intestinal damage following I/R. Furthermore, an imbalance in T helper type 1/2 cell (Th1/Th2) response may exacerbate necroptosis [61].

ROS‐driven thioredoxin‐interacting protein (TXNIP) dissociation serves as the key mechanism for NOD‐like receptor family, pyrin domain containing 3 (NLRP3) inflammasome assembly by mediating the recruitment of apoptosis‐associated speck‐like protein containing a CARD (ASC) and pro‐caspase‐1. Activated caspase‐1 and ROS‐induced nuclear factor kappa‐light‐chain‐enhancer of activated B cells (NF‐κB) signalling collectively drive the maturation of IL‐1β and IL‐18, ultimately executing pyroptosis [55]. Mitochondrial dysfunction modulates NLRP3 recruitment to mitochondria and inflammasome activation, thereby regulating the initiation of pyroptosis [61].

Furthermore, ROS accumulation and lipid peroxidation are central steps in ferroptosis. When ROS generation overwhelms the cellular antioxidant defences, they oxidize polyunsaturated fatty acids within cellular and organellar membranes, generating lipid peroxides that propagate ferroptosis. This process may subsequently furnish nutrients for bacterial proliferation and mediate immune dysfunction [56]. In intestinal ischemia‐induced ischemia–reperfusion injury models, localized perimicrovascular infiltration of neutrophil extracellular traps (NETs) is markedly enhanced. This process impairs FUNDC1‐dependent mitophagic flux, compromising mitochondrial autophagy and quality control, thereby driving intestinal microvascular endothelial cell ferroptosis [62].

In recent years, emerging modalities of cell death have been identified across various animal models of intestinal injury. Erebosis, initially described by Ciesielski et al. in *Drosophila* intestinal epithelium, represents a novel cell death mode characterized by mitochondrial depletion and diminished cytoplasmic content within Ance‐positive enterocytes, ultimately disrupting intestinal homeostasis. Its underlying molecular pathway, however, remains to be defined [63]. PANoptosis, conceptualized in 2019 as an integrated cell death paradigm, is a lytic process mediated by caspases, receptor‐interacting protein kinase (RIPK), and uniquely orchestrated by the PANoptosome [64]. Although it contributes to host defence by clearing infected cells, it also paradoxically enhances bacterial immune evasion [65]. Recent investigations have delineated its pathophysiological relevance in gastrointestinal malignancies and ischemia–reperfusion injury, implicating multi‐pathway crosstalk and inflammatory microenvironment modulation [66, 67, 68]. In Salmonella infection models, the effector SopF was shown to target intestinal epithelial cells and orchestrate PANoptosis through PDK1‐RSK signalling [69]. Furthermore, Diosmin was found to attenuate inflammatory bowel disease via suppression of PANoptosis in intestinal epithelial cells and concomitant modulation of gut microbiota and metabolites [70]. Collectively, these studies underscore the mechanistic significance of PANoptosis in intestinal infection, although its precise regulatory role in sepsis‐induced intestinal injury remains incompletely understood.

### 2.3 Inflammatory Dysregulation

During sepsis, intestinal injury exhibits a biphasic characteristic of immune dysregulation, encompassing both ‘hyperinflammation’ and ‘immunosuppression’. These phases can coexist or occur sequentially. The intestine acts as both a ‘motor’ and an ‘amplifier’ in this process, where immunosenescence represents a central determinant of sepsis‐induced immune dysfunction and subsequent organ injury [71].

In the initial hyperinflammatory phase, recognition of PAMPs and DAMPs by TLRs and NOD‐like receptors (NLRs) on gut epithelial and resident immune cells stimulates a pronounced proinflammatory cytokine response (tumour necrosis factor‐alpha (TNF‐α), IL‐1β, IL‐6, etc.). This response propagates gut barrier dysfunction via TJ disruption and heightened permeability, facilitating bacterial translocation and instigating systemic inflammation [72].

By amplifying the early hyperinflammatory response, immunosenescence predisposes septic patients to late‐phase immunoparalysis after compensatory anti‐inflammatory activation. This immunosuppressive state is marked by substantial depletion of intestinal lymphocytes and dendritic cells, leading to severely compromised antigen presentation and impaired adaptive immune responses [73, 74]. Neutrophils—key effectors of innate immunity—during acute inflammation may undergo functional impairment through receptor downregulation. This occurs via heterologous (e.g., chemokine receptor) or homologous (e.g., formyl peptide receptor 1 (FPR1)) desensitization induced by DAMPs and microbial‐associated molecular patterns (MAMPs), potentially blunting their responsiveness to subsequent microbial challenges. The release of dysfunctional neutrophils alongside suppression of humoral immunity further contributes to sepsis‐associated immunoparalysis [75].

Sepsis‐induced lymphopenia is mediated by several key pathophysiological mechanisms: (1) widespread lymphocyte apoptosis. (2) Impaired thymic output, bone marrow regeneration, and homeostatic or antigen‐driven proliferative replenishment [76]. (3) innate immune dysregulation due to reductions in CD4^+^ T cells and B cells. Altered CD4^+^ T cell numbers, phenotypes, effector functions and the Th1/T‐regulatory (Th1/Treg) cell ratio significantly contributing to immune dysfunction [77]. (4) Diminished antigen presentation resulting from downregulation of tissue pattern‐recognition molecules, alongside compromised lymphocyte activation and differentiation; (5) upregulation of canonical inhibitory immune checkpoint receptors including programmed cell death protein 1, cytotoxic T‐lymphocyte‐associated protein 4, and T‐cell immunoglobulin and mucin domain‐containing‐3 [78]. (6) Elevated secretion of immunosuppressive cytokines (e.g., TGF‐β, IL‐10) by Tregs and myeloid‐derived suppressor cells (MDSCs), which obstruct lymphocyte proliferation. (7) Immunometabolic alterations, heterogeneity, and redistribution of lymphocytes themselves, contributing to lymphopenia [75, 79]. Sepsis‐induced lymphopenia is characterized by extensive adaptive immune cell apoptosis, diminished T cell diversity and expansion of immunosuppressive cell populations. Within the intestinal mucosa, a pronounced reduction in γδ T cells and loss of T cell receptor (TCR) diversity correlate with heightened disease severity and mortality [76].

During sepsis, macrophages transition from a pro‐inflammatory (M1) to an anti‐inflammatory (M2) phenotype, impairing pathogen clearance. Concurrently, gut dysbiosis reduces the production of immunomodulatory metabolites such as short‐chain fatty acids (SCFAs), further suppressing immune responses [80].

### 2.4 Intestinal Barrier and Permeability Disorders

The intestinal barrier constitutes a multilayered system—comprising mechanical, chemical, immune and biological components—that collectively safeguards against aberrant permeability and bacterial translocation. This integrated structure encompasses the surface mucus, the epithelial lining and the underlying basement membrane [81].

The intestinal physical barrier, composed of intact epithelial cells, constitutes the gut's primary defence line, the tight lipid bilayer of the epithelial brush border, and paracellular connections. Among these, the TJ is the most important cell connection, primarily including zonula occludens‐1 (ZO‐1), occludin, cadherin, claudin and β‐catenin. This barrier functions by blocking the passage of intraluminal bacteria and antigens into the lamina propria and circulation, thus preventing the induction of abnormal immune activation [8], and its levels are a useful biomarker, correlating with the functional status of the intestinal mechanical barrier. Claudins (Claudin 1–27), Crumbs3 (CRB3), and tight junction‐associated marvel proteins (TAMPs) are the backbone components of bicellular TJs. Most claudin proteins have barrier‐sealing properties, and their density changes with location and microbial concentration [58], whereas claudin‐2 can form paracellular cation and water channels. Its upregulated expression is an important cause of increased pore pathway permeability in sepsis and can also lead to reduced immune cells, increased IL‐17 release, and abnormal T cell activation [82]. Impaired TJ barrier integrity is a key driver of inflammatory bowel disease pathogenesis. Pro‐inflammatory cytokines, including IL‐1β, TNF‐α and interferon‐γ, are critical regulators of TJ function. Specifically, IL‐1β enhances TJ permeability via NF‐κB activation, MLCK gene induction and microRNA‐mediated suppression of ZO‐1 transcription [83]. Mucosal damage caused by ischemia–reperfusion injury involves activation of intramucosal neutrophils, villous contraction and epithelial sloughing. Occlusion of the small intestinal mesenteric vasculature for 60 min induces the loss of the apical third of the villus epithelium, whereas ischemia for 120 min causes near‐total epithelial denudation; additionally, increased bile salt concentration can enhance mucosal permeability and electrolyte transport, alter TJ integrity, reversibly increase epithelial permeability, reduce sodium absorption, and stimulate electrogenic Cl^−^ secretion [84].

Digestive juices, enzymes and mucopolysaccharides secreted by the gastrointestinal tract, together with bacteriostatic substances produced by the normal intestinal microbiota, collectively form the intestinal chemical barrier [85]. Mucins (MUC) secreted by goblet cells polymerize to form the gel‐like intestinal mucus layer, providing an ecological niche that competitively binds bacterial adhesion sites with intestinal epithelial cells, retaining bacteria within the mucus layer and promoting their clearance through peristalsis [86]. Furthermore, antimicrobial peptides (AMPs) secreted by Paneth cells and intestinal alkaline phosphatase (IAP) produced by IECs are also important components of the mucus layer. MUC acts as a mucus trap, enhancing immune cell release, and exerts post‐toxic effects such as bactericidal/bacteriostatic activity, inhibition of LPS‐induced inflammatory cascades, and macrophage autophagy [8]. Additionally, sodium‐hydrogen exchanger 3 (NHE3) is a key mediator of electroneutral Na^+^/H^+^ exchange in the intestine, maintaining intestinal electrolyte homeostasis. Its impairment may be a significant cause of chronic inflammation‐related and infectious diarrhoea. Pro‐inflammatory agents and pathogenic bacteria can inhibit its expression and activity, induce interferon‐γ (IFN‐γ) expression, alter the adhesive strength of tight junction proteins and thereby affect paracellular permeability. Glucocorticoids or sodium butyrate (NaB) can antagonize these effects [7].

The commensal intestinal microbiota constitutes a biological barrier against pathogens and establishes baseline immune adaptation through mechanisms such as competition for nutrients and spatial niches, local acidification, and activation of AMPs, MUC and sIgA [8]. In healthy individuals, the gut is dominated by Firmicutes and Bacteroidetes, which together account for over 90% of total bacterial abundance. Bacteriocin‐producing *Bifidobacterium* and *Lactobacillus* species inhibit pathogenic bacteria and contribute to intestinal homeostasis. Sepsis induces severe gut dysbiosis, particularly marked by expansion of *Proteobacteria* and other Gram‐negative (G^−^) species [27]. In clinical studies involving preterm infants with sepsis, the Shannon diversity index of the gut microbiota is significantly reduced and negatively correlates with white blood cell count, blood lactate levels and length of ICU stay. The combined abundance of Firmicutes and Bacteroidetes can fall below 10%, accompanied by an altered Firmicutes/Bacteroidetes (F/B) ratio. Increased abundance of opportunistic pathogens such as *Enterococcus*, along with a marked reduction in SCFA‐producing genera such as *Anaerobutyricum*, are associated with microbiota dysfunction and poor prognosis in sepsis [3, 8]. Probiotics may modulate the gut microbiota by targeting PPARγ [87]. In preterm infants, gut microbial development is characterized by retardation and deficiency in D‐leucyl endopeptidase. Supplementation with *Enterococcus* strains producing this enzyme or with *Limosilactobacillu* species may activate the NOD2 receptor via muramyl dipeptide (MDP), thereby regulating macrophage polarization, suppressing hyperinflammation and reducing the risk of late‐onset sepsis (LOS) [88].

Inappropriate use of antibiotics is a major cause of gut dysbiosis and multidrug resistance. Clinical studies indicate that early in the course of antibiotic therapy, the α‐diversity of the intestinal microbiota is significantly lower than in non‐sepsis patients, with a trend towards structural homogenization [88]. Vancomycin may affect the phenotype and interaction of intestinal neurons and macrophages, alter the microbiome during intestinal development in preterm infants and increase the risk of complications [89]. Furthermore, current guidelines recommend against empirical coverage of multidrug‐resistant (MDR) pathogens and anaerobes in the absence of confirmed infection. In mechanically ventilated patients with a low resistance background, selective digestive decontamination (i.e., topical antibiotics in the oropharynx and upper digestive tract combined with short‐term broad‐spectrum intravenous anti‐infective therapy) may be considered [34]. Other interventions, including enteral nutrition, proton pump inhibitors and catecholamines, can also alter the gut microbial environment. Intestinal ischemia–reperfusion (I/R) triggers mucosal inflammation, promoting overgrowth of Proteobacteria and G^−^ bacteria. Supplementation with probiotics or specific metabolites may alleviate dysbiosis and reduce bacterial translocation through immunomodulatory mechanisms [26].

The intestinal immune barrier, often regarded as a ‘second line of defence’, is orchestrated by the gut‐associated lymphoid tissue (GALT). Its activation involves a Th1‐polarized cytokine response from lymphocytes targeting infected or stressed epithelial cells, alongside the stimulated secretion of SIgA. This initiates a humoral immune response to prevent pathogenic bacterial adhesion and colonization on the mucosal surface [8, 90]. The functional integrity of this barrier is critically dependent on lamina propria macrophages and the composition of T‐cell subsets within mesenteric lymph nodes [9, 10]. The excessive immune response and immunoparalysis during sepsis are significant causes of severe tissue injury [71].

### 2.5 ISCs and Tissue Repair Mechanisms

During infection, ISCs‐driven epithelial renewal is critical for mucosal repair, wound closure, and containment of systemic infection. Residing in the Lieberkühn crypts, ISCs differentiate into transit‐amplifying (TA) cells, thereby giving rise to the diverse epithelial lineages—including absorptive enterocytes, enteroendocrine cells (EECs), and secretory Paneth and goblet cells [91]. This process is precisely orchestrated by Wnt/β‐catenin signalling and pro‐stemness factors that maintain ISCs' function, whereas bone morphogenetic proteins (BMPs) drive differentiation along the crypt‐villus axis [91, 92]. In the absence of Paneth cells, deep crypt secretory cells compensate by maintaining ISCs' function through Notch signalling [58].

Following acute intestinal mucosal injury induced by ischemia or luminal injury, neutrophils are recruited to the lamina propria, where they release signals that facilitate tissue repair—including prostaglandin E_2_ and cyclooxygenase‐2 (COX‐2)—that initiate the healing process [84]. This response involves the activation of subepithelial myofibroblasts and villous contraction. Re‐epithelialization is subsequently driven by epithelial cell migration via focal adhesion kinase, growth factor, and integrin‐mediated signalling, ultimately restoring mucosal continuity [84]. Re‐establishment of TJs and paracellular sealing represents a critical final step in mucosal repair. Under physiological conditions, this coordinated mechanism enables rapid wound closure, thereby preventing bacterial transmigration and the onset of sepsis [84]. Concurrently, immune cells, cytokines, and associated signalling molecules remodel the niche microenvironment surrounding ISCs, thereby facilitating ISC proliferation and differentiation to promote epithelial repair [93].

The unique capacity of ISCs to proliferate and differentiate has catalyzed the revolutionary emergence of organoid‐based three‐dimensional therapeutic platforms. Under defined conditions with key signals such as Wnt, R‐spondin, and Noggin, a single Lgr5^+^ ISC can self‐organize into a three‐dimensional ‘intestinal organoid’, thereby underscoring its value in disease modelling, drug screening, and regenerative medicine applications [94, 95]. Under inflammatory stress, Lgr5^+^ ISCs undergo metabolic reprogramming, leading to the accumulation of succinate and epigenetic remodelling. Even after inflammation resolves, these ISCs retain functional impairment, exhibiting reduced differentiation and regenerative capacity in organoid cultures [94, 96]. This cell‐intrinsic inflammatory memory, stably transmitted through ISCs, is characterized by increased chromatin accessibility and altered chromatin states at inflammation‐related loci, contributing to persistent immune dysregulation [96, 97].

### 2.6 Gut‐Target Organ Axis

The gut‐target organ axis orchestrates the pathogenesis and progression in sepsis. As a major interface between the host and the external environment, the intestine engages in extensive bidirectional crosstalk with remote organs via the circulatory and lymphatic systems. Anatomically, the gut and liver are interconnected via the portal vein, constituting the ‘gut‐liver axis’ [8, 98]. This connection allows for the direct hepatic delivery of gut‐derived metabolites and microbial products, while the liver, in turn, regulates the intestinal environment through the secretion of bile acids and immunoglobulins [8, 98]. In sepsis, gut barrier failure drives the systemic dissemination of microbial products, which amplifies the inflammatory response, culminating in a vicious cycle of distal organ injury [99]. The liver serves as a central hub for host defence by clearing pathogens, producing acute‐phase reactants and regulating inflammatory mediator release [99]. Furthermore, gut microbiota‐derived metabolites are critical for maintaining immune homeostasis and barrier integrity. Notably, primary bile acids are metabolized by the microbiota into secondary bile acids, which reinforce intestinal barrier function through farnesoid X receptor (FXR) activation and attenuate sepsis‐induced injury [98].

The gut‐brain axis (GBA) serves as a dynamic, bidirectional communication network connecting the gut and the brain. This interaction is mediated through the regulation of Treg cell differentiation, metabolite‐driven anti‐inflammatory responses and modulation of the enteric nervous system (ENS) [100, 101]. A principal clinical manifestation of this dysregulation is sepsis‐associated encephalopathy (SAE) [102], an acute cognitive impairment secondary to peripheral infection and a common early sign of organ dysfunction. The SAE pathogenesis is closely linked to intestinal barrier failure, microbial translocation and subsequent disruption of GBA signalling [101]. Specifically, the translocation of gut‐derived toxins and inflammatory mediators across the impaired blood–brain barrier drives glial hyperactivation, neuroinflammation and eventual cognitive impairment [103]. The microbiota‐gut‐brain axis functions via neural, endocrine, and immune pathways: (1) Neurally, acetylcholine signalling via the α7‐nicotinic acetylcholine receptor (α7nAChR) modulates neuron–glia crosstalk; (2) Endocrinally, hypothalamic–pituitary–adrenal (HPA) axis overactivation correlates with gut homeostasis disruption, while glucagon‐like peptide‐1 (GLP‐1) and vasoactive intestinal peptide (VIP) exert stage‐dependent regulatory effects; (3) Immunologically, Th17 cells—which help maintain gut immune homeostasis—can migrate to the central nervous system (CNS), where their interaction with microglial M1/M2 polarization imbalance contributes to blood–brain barrier dysfunction and SAE pathogenesis [101, 103]. Microbiota‐derived metabolites serve as key neuromodulators. SCFAs and indole‐3‐propionic acid (IPA) exhibit neuroprotective effects by regulating the NLRP3 inflammasome [104]; while microbial tryptophan and glutamate influence central serotonin levels [75]. Experimental studies show that diminazene aceturate (DIZE) increases CNS serotonin while suppressing splenic TNF production [105]; and non‐hepatic hyperammonemia (NHH) in septic mice leads to hippocampal ammonia accumulation, potentially upregulating astrocytic aquaporin‐4 (AQP4) to facilitate SAE development [106]. The amelioration of cognitive deficits in murine SAE models by strategies targeting gut barrier function and microbial ecology underscores their therapeutic potential [107, 108], although the precise mechanisms of glial‐cell and microbiota crosstalk warrant further investigation.

The gut‐lung axis represents a crucial immunoregulatory circuit connecting intestinal and pulmonary immunity, providing compelling support for the ‘gut‐lymph’ theory of pathological crosstalk [109]. This communication is partly mediated by the shared mucosal immune system, whereby gut microbiota composition directly shapes pulmonary immunity [26]. For instance, intestinal dysbiosis can stimulate the recruitment of γδ T17 cells to the lung, instigating an IL‐17A‐driven inflammatory cascade that exacerbates lung injury [110]. Notably, faecal microbiota transplantation has been shown to restore microbial homeostasis and confer protection against pneumococcal pneumonia by enhancing alveolar macrophage function, elevating levels of beneficial metabolites such as SAFC and secondary bile acids, and improving clinical outcomes while reducing antibiotic resistance [11, 109]. Similarly, the gut‐kidney axis plays a significant role in sepsis progression. Intestinal barrier disruption promotes the systemic dissemination of bacteria and toxins, exacerbating acute kidney injury via STING pathway activation and NLRP3 inflammasome [111, 112].

**FIGURE 2:**
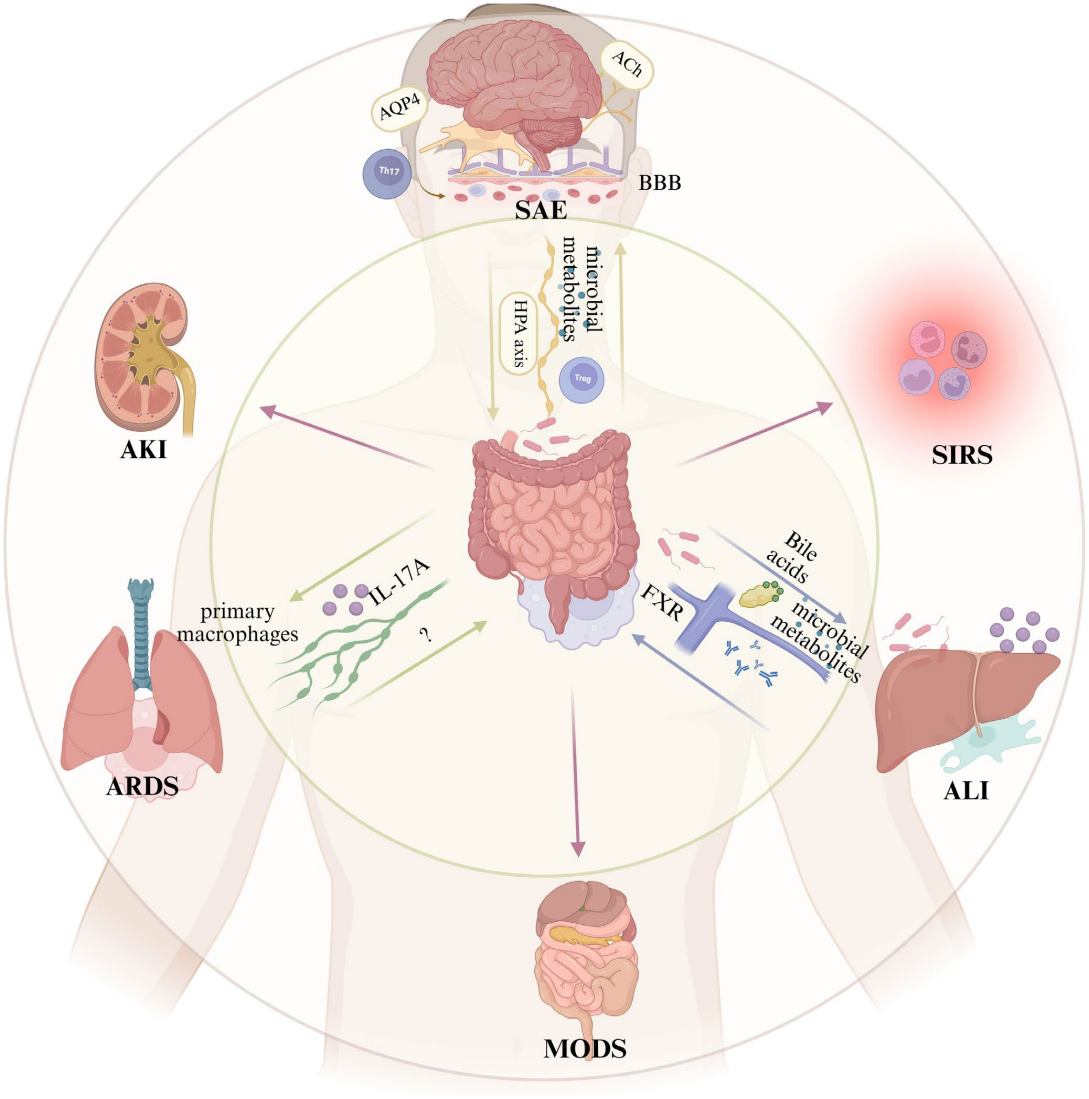
Gut‐target organ axis. The gut acts as a central driver of sepsis, influencing remote organs through a complex multi‐pathway network. The microbiota‐gut‐brain axis modulates blood–brain barrier integrity and the development of sepsis‐associated encephalopathy via neuroendocrine–immune interactions. The gut–liver axis engages in bidirectional crosstalk through the portal vein and bile acid–FXR receptor pathways, coordinately regulating immune and metabolic homeostasis. The gut–lung axis primarily facilitates the delivery of inflammatory mediators via the intestinal lymphatic circulation, driving acute lung injury. Concurrently, intestinal dysbiosis and disruption of the gut barrier promote systemic inflammatory response, exacerbate acute kidney injury through circulating factors and ultimately contribute to the pathogenesis of multiple organ dysfunction syndrome.

Figure 2 synthesizes the bidirectional crosstalk of the gut‐target organ axis and its key pathways, highlighting the role of intestinal injury in the systemic progression of sepsis. Through its intricate anatomical and physiological connections, the gut‐target organ axis functions as a critical amplifier of remote organ injury during sepsis. A deeper mechanistic understanding of the gut‐liver/brain/lung/kidney axes therefore unveils promising therapeutic avenues for sepsis management.

**FIGURE 3:**
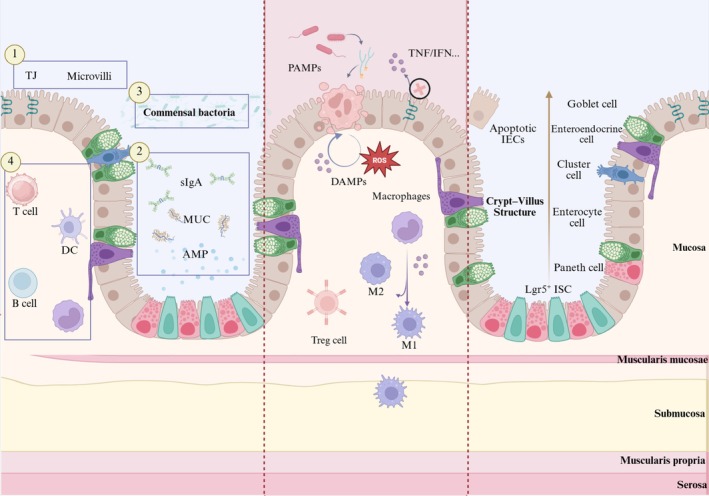
Pathophysiological features of sepsis‐induced intestinal injury. Under physiological conditions, intestinal epithelial cells work in concert with the commensal microbiota and their metabolites to maintain the gut barrier and intestinal immune homeostasis. The proliferative capacity and differentiation potential of intestinal stem cells are essential prerequisites for ensuring the continuous turnover of the intestinal epithelium and sustaining its diverse functional network, thereby enabling compensatory repair upon injury. However, during sepsis, the gut suffers a dual assault from both microbial dysbiosis and ischemia–reperfusion injury. This leads to a cascade of pathological alterations, including the accumulation of oxidative stress, endothelial dysfunction, disruption of the barrier integrity, dysregulated immune responses and impaired regenerative function of intestinal stem cells. Collectively, these changes drive bacterial translocation and fuel the systemic progression of sepsis.

Oxidative stress and dysregulated inflammation are pivotal drivers of intestinal injury. The pathophysiological basis of oxidative stress is established by ischemia‐hypoxia from systemic blood flow redistribution and subsequent reperfusion injury. Mitochondrial dysfunction, coupled with the activation of mitochondria‐endoplasmic reticulum contact sites, serves as a critical instigator of inflammatory signalling, pyroptosis and other PCD in IECs [20, 113]. These events culminate in intestinal endothelial dysfunction, downregulation of tight junction protein expression, impaired secretion of key protective factors (e.g., MUC, IAP, AMPs), aberrant macrophage polarization and disruption of the gut microbiota. The consequent collapse of the intestinal barrier and increased mucosal permeability facilitate bacterial translocation into the systemic circulation, thereby promoting the development of gut‐derived sepsis. Figure 3 summarizes the barrier function of the intestine and its role in injury repair, while illustrating the pathophysiological features of intestinal injury during sepsis. These interconnected pathological cascades are governed by complex molecular pathways [20].

## 3 Molecular Regulatory Mechanisms of Sepsis‐Induced Intestinal Injury

### 3.1 Oxidative Stress Disorder

Accumulation of ROS following I/R injury is a central factor in intestinal damage. Mitochondria serve as the primary sites for ROS processing; mitochondrial dysfunction arising from structural damage, dysregulated mitochondrial dynamics, and impaired autophagy constitutes a critical link in redox imbalance [43, 113, 114]. Under hypoxic conditions, intracellular pH decreases, and Na^+^‐H^+^ exchange leads to intracellular Na^+^ overload, which subsequently activates 2Na^+^‐Ca^2+^ exchange (NCX) and results in Ca^2+^ influx overload [113]. Upon reperfusion, the electron transport chain is reactivated, while xanthine oxidase and NADPH oxidase (NOX) family enzymes generate substantial amounts of ROS. These ROS induce opening of the mitochondrial permeability transition pore (MPTP), mediate neutrophil chemotaxis and sarcoplasmic reticulum dysfunction, and promote lipid peroxidation and enzyme denaturation. ROS accumulation also facilitates the release of DAMPs [115]. Ultimately, these events lead to imbalanced energy metabolism in IECs, cell death and activation of inflammatory signalling cascades [43, 114]. During septic intestinal injury, the mitochondrial inner membrane protein uncoupling protein 2 (UCP2) modulates these processes by regulating the mitochondrial membrane potential (proton motive force, Δp) [116]. UCP2 deficiency amplifies oxidative stress and inflammation, marked by elevated ROS and malondialdehyde (MDA) alongside diminished superoxide dismutase (SOD) and glutathione peroxidase (GPX) activity. This redox dysregulation further skews cytokine production towards a pro‐inflammatory profile, suppressing anti‐inflammatory mediators including IL‐10 [117].

Nuclear factor erythroid 2‐related factor 2 (Nrf2), a master regulator of the antioxidant response, is constitutively inhibited by Keap1, which promotes its ubiquitination and proteasomal degradation [118]. The Nrf2‐Keap1 axis represents a pivotal signalling pathway for mitigating oxidative stress during sepsis. Upon activation, Nrf2 is released from Keap1, undergoes nuclear translocation, and binds the antioxidant response element (ARE), thereby driving the expression of cytoprotective genes [117]. This transcriptional program upregulates a spectrum of antioxidants, including heme oxygenase‐1 (HO‐1) and SOD, thereby enhancing the total antioxidant capacity (T‐AOC) and reducing levels of the lipid peroxidation marker MDA [20, 55, 115]. Furthermore, through its modulation of GPX activity, the Nrf2‐GPX4 axis can inhibit ferroptosis, a mechanism implicated in sepsis‐induced liver and lung injury [119, 120], though its intestinal functions remain incompletely characterized. Given its upstream regulatory position, Nrf2 activation status serves as both a key indicator of systemic oxidative stress and a therapeutic target for intervention in sepsis.

### 3.2 Cytokine Storm and Immunoparalysis

Sepsis‐induced intestinal injury activates multiple inflammatory pathways, notably NF‐κB and GMP‐AMP (cGAMP) synthase (cGAS)‐STING, initiating a cascade that amplifies inflammatory signals and culminates in a cytokine storm. The hyperactivation of the NF‐κB subunit p65 is a central event in this dysregulated immune response [121]. Under physiological conditions, NF‐κB activity is restrained by inhibitor of kappa B (IκB), which is phosphorylated by the upstream IκB kinase (IKK) complex. A feedforward loop exists between ROS and NF‐κB signalling, mutually reinforcing their activation during sepsis [122]. TLR4 on epithelial and immune cells serves as the primary receptor for LPS, mediating much of the ensuing intestinal injury [123]. TLR4 activates NF‐κB through two principal pathways: the MyD88‐dependent cascade inducing NF‐κB and activator protein 1 (AP‐1), and the TRIF‐dependent cascade activating interferon regulatory factor 3 (IRF3) and NF‐κB [124]. This results in the robust production and systemic release of pro‐inflammatory cytokines, including IL‐1β, IL‐6 and IL‐18. The gut microbiota‐derived metabolite NaB can attenuate inflammation by inhibiting NF‐κB p65 nuclear translocation and stabilizing IκBα [121]. Paradoxically, however, specific inhibition of IKKβ in intestinal epithelial cells can exacerbate injury, suggesting a context‐dependent, IKKβ‐mediated signalling exerts a protective function in the intestinal mucosa [125].

The second messenger cGAS and the adaptor STING constitute the cGAS‐STING pathway, a cornerstone of the innate immune system, functioning as a critical mediator of acute intestinal I/R injury. Its significance is underscored by elevated STING expression observed in both septic models and human intestinal tissues, with pathway activation potentially initiated by bacterial components [126]. The cGAS‐STING pathway initiates a signalling cascade in response to aberrant DNA: cGAS produces cGAMP, activating STING, which then traffics to the Golgi and recruits TANK‐binding kinase 1 (TBK1) to phosphorylate IRF3 and promote NF‐κB signalling. This cascade drives the production of type I interferons (e.g., IFN‐β) and pro‐inflammatory cytokines like TNF‐α, thereby instigating a potent inflammatory response and promoting intestinal barrier failure [126, 127]. Other signalling pathways also contribute to intestinal inflammation. The Notch3‐Smad axis has been implicated in promoting intestinal inflammation and fibrosis [22]. Furthermore, activated neutrophils extrude neutrophil extracellular traps (NETs)—decondensed chromatin webs decorated with granular proteins—via a peptidylarginine deiminase 4 (PAD4)‐dependent process. PAD4 promotes NET release via histone citrullination‐driven chromatin decondensation. Liberated NETs perpetuate inflammation by activating TLR9‐mediated ER stress, the C/EBP homologous protein (CHOP) pathway and ROS‐dependent signalling [128].

The intestinal lamina propria maintains a diverse repertoire of immune cells critical for innate immune homeostasis. Among these, gut‐resident macrophages (gMacs) play a central role, characterized by high expression of chemokines such as CX3CL1 and its receptor CX3CR1. Activation of the TAK1/p38MAPK/MK2 signalling axis in macrophages releases pro‐inflammatory cytokines, including IL‐6, TNF‐α and enhances chemokine expression [10, 129]. Macrophages located predominantly in the submucosa can undergo dynamic polarization into M1/2 phenotypes. Promoting macrophage proliferation and M2 polarization represents a potential therapeutic strategy for mitigating intestinal inflammation [130]. The transcription factor activating transcription factor (ATF), a bZIP superfamily protein, is critical for macrophage differentiation. During sepsis, TGF‐βR activation downregulates ATF, resulting in an imbalance of macrophage subsets and impaired differentiation [10]. The macrophage surface receptor Mertk stimulates M2 macrophage polarization via the Mertk‐signal transducer and activator of transcription 1 (STAT1)/SOCS pathway and clears apoptotic cells through Protein S (PROS1) [130]. In alveolar macrophages, Wnt signalling activation drives C‐C motif chemokine ligand 1 (CCL1) upregulation, promoting γδ T17 cell migration and expansion of specific CD44^+^ Ly6C^+^ IL‐7R^+^ CD8ˡ°ʷ populations, whereas esketamine attenuates acute lung injury (ALI) via suppression of Wnt/β‐catenin‐driven pulmonary inflammation [110]. Fang Tan et al. also found that under diabetic conditions, upregulation of Snail exacerbates oxidative stress and inflammation in a manner correlated with macrophage infiltration. miR‐3061 appears to counteract this effect by targeting Snail, thereby inhibiting M1 polarization and inflammatory factor release [131]. Mitochondria are also key participants in innate and adaptive immunity. LPS/IFN‐γ suppresses PTEN‐induced putative kinase 1 (PINK1)‐dependent mitophagy in macrophages via STAT1‐mediated caspase‐1/11 activation, while promoting classical macrophage polarization in an mtROS‐dependent manner [132]. Furthermore, an increased proportion of Treg cells in mesenteric lymph nodes (MLNs) and upregulation of forkhead box P3 (FoxP3) and CTLA‐4 expression can enhance the immunoregulatory capacity of Treg cells, achieving an anti‐inflammatory effect [9].

**FIGURE 4:**
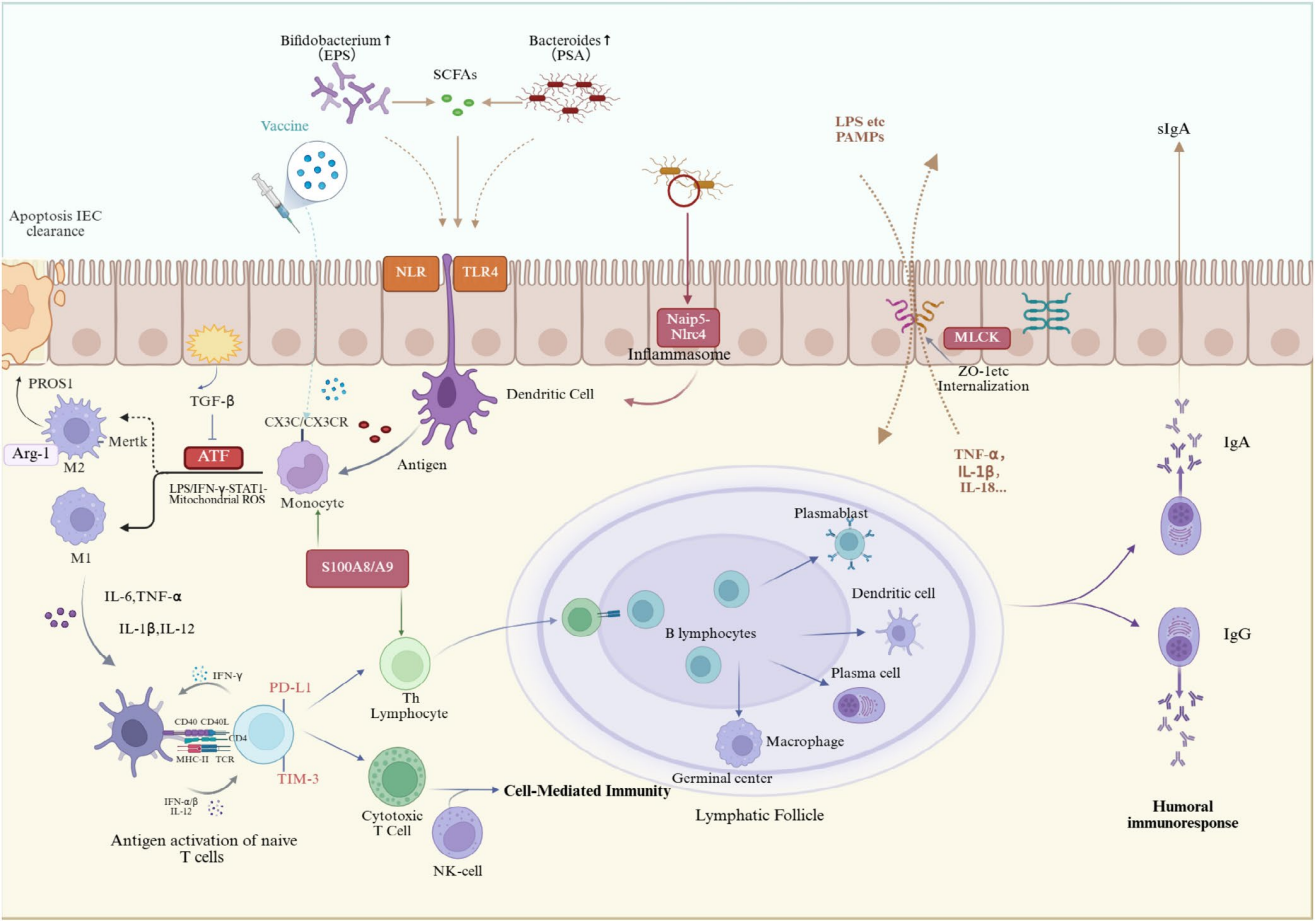
The immune dysregulation mechanism in sepsis‐induced intestinal injury. This diagram delineates the biphasic dysregulation of the immune network during sepsis‐induced intestinal injury. The initial hyperinflammatory phase: Microbial components (e.g., LPS, flagellin) activate immune cells via pattern recognition receptors (TLRs/NLRs), triggering NLRP3 inflammasome assembly and massive release of IL‐1β and IL‐18; a storm of proinflammatory cytokines (TNF‐α, IL‐6) disrupts intestinal epithelial tight junctions (e.g., ZO‐1 internalization), exacerbating barrier damage. The subsequent immunosuppressive phase: persistent activation induces lymphocyte (particularly CD4+ T cell) apoptosis and dendritic cell exhaustion, accompanied by a compensatory anti‐inflammatory response (e.g., TGF‐β upregulation), ultimately leading to dual paralysis of cellular and humoral immunity (impaired germinal centre formation), characteristic of sepsis‐induced ‘immune paralysis’.

Sepsis‐induced immunosuppression represents a critical facet of immune dysregulation, characterized by impaired humoral immunity. This is evidenced by the depletion and functional attenuation of germinal centre T follicular helper (Tfh) CD4^+^ T cells and B cells, coupled with a sharp decline in circulating immunoglobulin M (IgM) levels. These deficits heighten susceptibility to secondary infections and viral reactivation [133, 134]. Our preliminary research has revealed that nuclear FMR1 interacting protein 1 (NUFIP1)‐mediated ribophagy exerts a protective effect against apoptosis in CD4^+^ T cells. Specifically, sepsis‐induced ribophagy activates the Z‐DNA binding protein 1 (ZBP1)‐mediated cGAS‐STING signalling pathway, recruiting NUFIP1 into the STING protein complex. This process mitigates PANoptosis in CD4^+^ T lymphocytes during septic conditions and alleviates immune paralysis [135]. Jorge D. et al. discovered that the Itaconate Pathway is a central regulatory node for immunological tolerance. β‐glucan reprograms monocyte function by modulating immunometabolic pathways: it suppresses immune‐responsive gene 1 (IRG1)/itaconate synthesis while enhancing succinate dehydrogenase (SDH) activity to promote the tricarboxylic acid (TCA) cycle, thereby reversing innate immune tolerance and inducing trained immunity [136]. Mitochondrial uncouplers that potentiate mitophagy can reverse LPS/IFN‐γ‐mediated macrophage activation, which leads to immunoparalysis and impaired bacterial clearance. Inhibiting mitophagy may improve the state of immunoparalysis in sepsis and enhance prognosis [132]. Calprotectin (S100A8/A9), an endogenous TLR4 ligand and DAMP protein, promotes microbial stress tolerance while functioning as a clinical biomarker for acute inflammation. S100A8/A9 can induce a regulatory phenotype in lamina propria macrophages, facilitating Treg cell expansion. While highly expressed in breast milk and important for neonatal intestinal immunity, its precise function in sepsis‐induced intestinal injury awaits further clarification [137]. Precision immunotherapy—aimed at attenuating hyperinflammation, reversing immunoparalysis, and modulating vascular tone—has emerged as a strategic focus in sepsis management. However, the efficacy of such interventions specifically for septic intestinal injury awaits validation [138]. Figure 4 summarizes the key mechanisms underlying dysregulated intestinal immune cell responses in sepsis. These alterations collectively contribute to both early local hyperinflammation and subsequent immunosuppression.

### 3.3 Dysregulated Programmed Cell Death

The accrual of oxidative stress and inflammatory mediators functions as DAMPs, triggering inflammatory cell death in intestinal epithelial cells—predominantly pyroptosis and necroptosis. ROS promote the assembly of the NLRP3 inflammasome by inducing TXNIP (thioredoxin‐interacting protein) dissociation, facilitating the recruitment of ASC and pro‐caspase‐1, which in turn activates caspase‐1 and its downstream cascade [55]. Concurrently, ROS activate the NF‐κB pathway, enhancing IL‐1β/IL‐18 maturation and driving pyroptosis [55]. TNF and IFN can also induce gasdermin D (GSDMD) cleavage via caspase‐11, mediating pyroptosis in both macrophages and epithelial cells. Mitochondrial dysfunction, including dysregulated dynamics, is a key activator of the inflammasome [109, 139]. This involves upregulation of mitofusin 2 (MFN2), a core protein at mitochondria‐endoplasmic reticulum contact sites (MAMs), where NLRP3 localizes to facilitate inflammasome activation [140]. Conversely, downregulation of the mitochondrial uncoupling protein UCP2 exacerbates oxidative stress and promotes NLRP3‐mediated pyroptosis, an effect reversible by the NLRP3 inhibitor MCC950 [117].

C‐terminus of HSC70‐interacting protein (CHIP), acting as an E3 ubiquitin ligase, degrades proteins via the ubiquitin‐proteasome system. It degrades Karyopherin Subunit Alpha 2 (KPNA2) to inhibit NF‐κB nuclear translocation, and degrades extracellular signal‐regulated kinase (p‐ERK) to suppress ERK‐AP‐1 (c‐Fos/c‐Jun dimer) activation, thereby downregulating TNF‐α and IL‐1β transcription. The compound exerts a direct inhibitory effect on the NF‐κB/NLRP3 inflammasome pathway, leading to the downregulation of key components including NLRP3, cleaved caspase‐1 and GSDMD‐NT, which consequently suppresses pyroptosis [141, 142]. Separately, the adaptor protein Card9 competitively binds caspase‐1 in a RIPK2‐dependent manner, impairing NLRP3 inflammasome assembly and specifically reducing pro‐IL‐1β transcription [143].

In parallel, ROS acts as a trigger for RIPK1/RIPK3‐dependent necroptosis through the phosphorylation and membrane translocation of mixed lineage kinase domain‐like protein (MLKL), initiating MLKL‐mediated necroptosis. Meanwhile, RIPK3 sustains STING signalling by suppressing its autophagy through the protein kinase‐ Unc‐51 like autophagy activating kinase 1 (AMPK‐ULK1) pathway. Furthermore, upon recognition of cyclic dinucleotides or cytosolic DNA, activated STING translocates from the endoplasmic reticulum to the Golgi apparatus, where it recruits TBK1 and IRF3 to initiate IFN release and inflammatory responses [144, 145].

LPS also initiates signalling such as TNF or regulates pathways like Wnt, phosphatidylinositol 3‐kinase/AKT serine/threonine kinase/mechanistic target of rapamycin (PI3K/Akt/mTOR), and Janus kinase (JAK)/STAT to modulate apoptosis and autophagy [20, 55, 115, 146]. Among these, tumour necrosis factor receptor 1 (TNFR1) internalization is a key signal inducing IECs' apoptosis. The membrane protein EF‐Hand domain family member D2 (EFHD2) can inhibit TNFR1 internalization by suppressing Cofilin phosphorylation, thereby inhibiting IECs' apoptosis and exerting an anti‐inflammatory effect [147]. Under physiological conditions, IECs maintain survival signals through integrin‐mediated adhesion to the extracellular matrix (e.g., laminin, collagen), which induces focal adhesion kinase (FAK) and Src family kinases, subsequently promoting pro‐survival PI3K/AKT and Ras/ERK signalling [146]. Upon detachment from the basement membrane, integrin clustering is disrupted, leading to the inactivation of FAK and its downstream pathways. This cascade triggers apoptosis through the downregulation of anti‐apoptotic proteins and upregulation of pro‐apoptotic factors, leading to increased mitochondrial outer membrane permeability (MOMP), cytochrome c release, and caspase cascade activation [146]. While IECs death can occur via apoptosis, autophagy and necroptosis, IL‐9 from macrophages disrupts intestinal integrity by elevating transferrin receptor, Fe^2+^, and iFABP, and suppressing glutathione (GSH), GPX4 and ZO‐1. That ferroptosis inhibition normalizes these changes strongly suggests its involvement in IL‐9‐driven injury [148], with Nrf2 potentially playing a regulatory role in this process [149].

**FIGURE 5:**
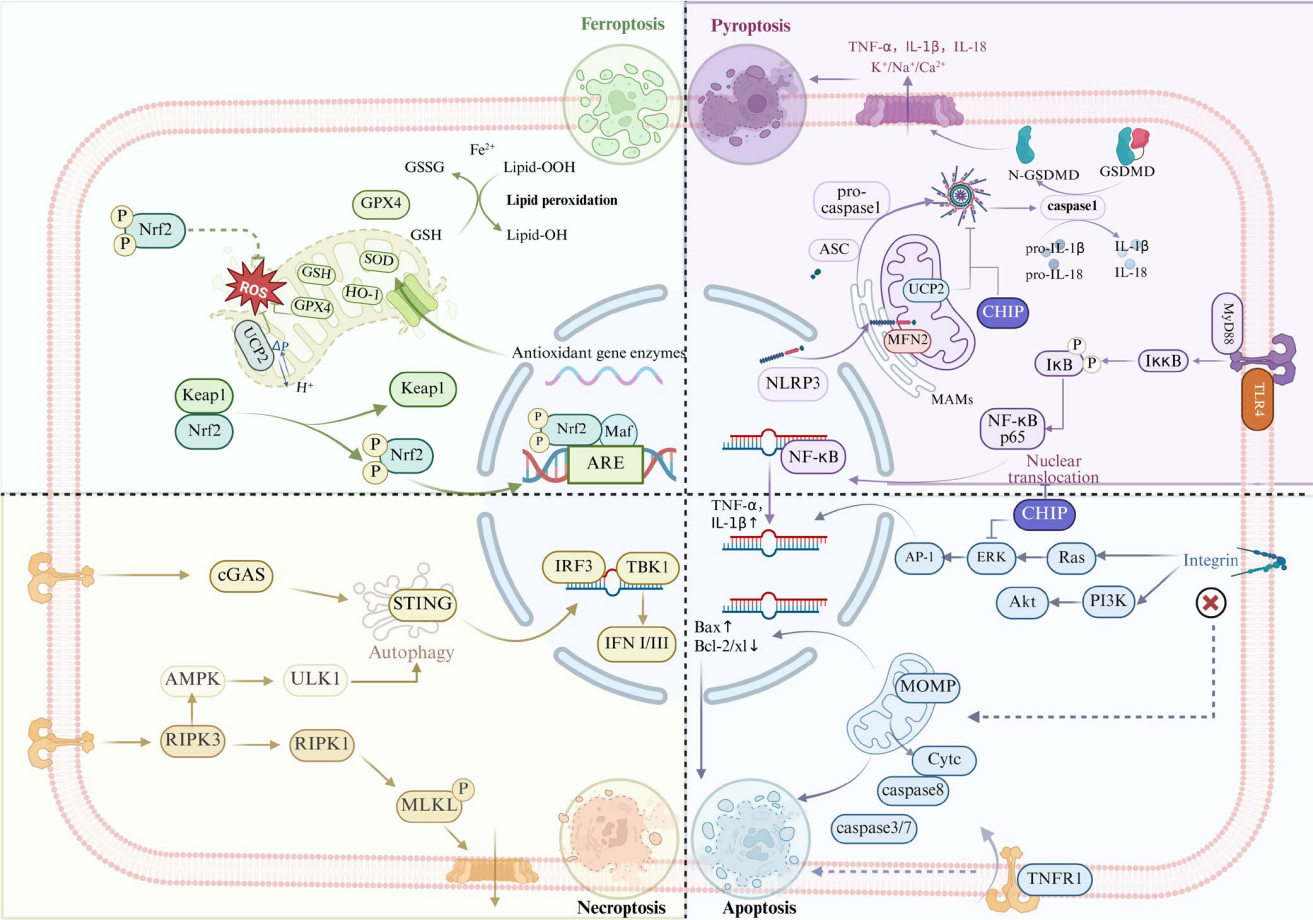
Molecular pathways of intestinal epithelial cell dysfunction in sepsis. This diagram illustrates the intricate molecular network of programmed cell death in intestinal epithelial cells during sepsis‐induced intestinal injury. Stimulated by PAMPs and DAMPs, multiple upstream pathways are concurrently activated: Nrf2‐Keap1 system disruption exacerbates oxidative stress; the NF‐κB pathway drives inflammatory cytokine release; cGAS‐STING signalling initiates innate immune activation; and mitochondrial dysfunction (manifested as membrane potential collapse and MAMs dysregulation) amplifies cellular stress. These signals subsequently orchestrate distinct downstream death programs: GPX4‐GSH axis failure induces ferroptosis; caspase‐1‐mediated GSDMD cleavage executes pyroptosis; and the RIPK1/RIPK3/MLKL pathway mediates necroptosis. Significant crosstalk exists among these pathways, forming positive feedback loops that collectively compromise IEC barrier function and drive sepsis progression.

Crosstalk between different forms of PCD significantly influences IEC fate. For instance, TNF and IFN signalling mediate injury crosstalk in target organs like the small intestine. Autoactivation of pro‐apoptotic caspase‐8 synergizes with the caspase‐11‐GSDMD non‐canonical inflammasome to trigger endotoxemia. TNF induces caspase‐8 auto‐processing, while IRF3‐dependent signalling induces IFNβ and Casp11; binding to interferon alpha and beta receptor subunit 1 (IFNAR1) mediates extrinsic cell death and drives ileal injury [59]. Figure 5 illustrates the key mechanisms of sepsis‐induced intestinal injury, wherein multiple forms of PCD in IECs drive dysfunction through interplay with oxidative stress, inflammation and inter‐PCD crosstalk.

A broader integrative concept, PANoptosis, a recently proposed concept of regulated cell death, can simultaneously trigger multiple cell death outcomes. IFN‐γ induces enterocyte PANoptosis, and treatment with a selective JAK1 inhibitor can benefit patients with Crohn's disease [150].

### 3.4 Microbiome Dysbiosis and Translocation

Microbial dysbiosis and subsequent bacterial translocation, leading to an increased load of pathogenic bacterial components, serve as direct triggers of localized infections. TLRs function as the primary sensors for injury induced by PAMPs [151]. Nevertheless, the specific signalling pathways that modulate microbiota composition, barrier integrity, and the systemic immune response to translocated bacteria remain incompletely characterized [152]. A seminal study by Janelle S. Ayres and colleagues demonstrated that antibiotic‐induced intestinal injury in mice can precipitate a lethal, sepsis‐like syndrome, driven by the systemic expansion of multidrug‐resistant *Escherichia coli* [153]. This pathology is mediated by the Naip5‐Nlrc4 inflammasome, which detects bacterial flagellin translocated into the host cytosol via specialized secretion systems. These findings indicate that dysbiosis and loss of intestinal homeostasis can provoke aberrant innate immune signalling and rapid mortality, positioning the Naip5‐Nlrc4 axis as a potential therapeutic target for infections involving antibiotic‐resistant pathogens [153].

The gut microbiota‐derived metabolite minaprine (MC) confers protection against intestinal ischemia/reperfusion injury in a manner dependent on the aryl hydrocarbon receptor (AhR)/group 3 innate lymphoid cells (ILC3)/IL‐22 signalling axis [154]. Furthermore, enterobacterial metabolites activate macrophage‐specific AhR, modulating its transcriptional reprogramming [155].

As pivotal effector cells in intestinal immunity, macrophages are a critical component of the innate defence against the gut microbiota. The endogenous TLR4 ligand calprotectin (S100A8/A9) can modulate the phenotype of lamina propria macrophages and influence Treg cell abundance, correlating with an increased faecal abundance of Enterobacteriaceae. This positions calprotectin as a key mediator of neonatal gut microbial colonization and immune development [137].

### 3.5 Abnormal Proliferation, Differentiation, and Tissue Repair of ISCs

During sepsis, the proliferative, differentiation, and reparative capacities of ISCs are significantly suppressed. Although research into the underlying mechanisms remains relatively limited, recent studies have provided deeper insights. ISC growth factors bind to the Lgr5^+^ receptor and stimulate extracellular vesicle‐mediated Wnt/β‐catenin signalling, facilitating β‐catenin nuclear translocation and driving the proliferation and regeneration of Lgr5^+^ ISCs for epithelial repair. Dickkopf‐1 (DKK1), a negative regulator of this pathway, may contribute to intestinal injury—such as that induced by radical chemoradiotherapy for gastrointestinal malignancies—through its aberrant expression [156]. Upregulated in intestinal crypt cells after I/R injury, plasma exosomal circEZH2_005 alleviates mucosal damage by promoting Lgr5^+^ stem cell proliferation via direct interaction with hnRNPA1 and enhanced Gprc5a stability, highlighting its therapeutic potential [157].

Lamina propria macrophage‐derived Lactadherin (milk fat globule‐EGF factor 8, MFG‐E8) promotes mucosal healing by binding phosphatidylserine at wound edges via a PKCε‐dependent mechanism, triggering F‐actin cytoskeletal reorientation in intestinal epithelial cells. MFG‐E8 also facilitates cell migration and tissue remodelling through interactions with αvβ3/αvβ5 integrins. Its reduction during sepsis impairs wound closure [91]. X‐Box binding protein 1 (XBP‐1) is a central component of the Ire1α/XBP‐1 branch, which comprises one of the three major pathways of the unfolded protein response (UPR) to endoplasmic reticulum stress. Sepsis‐upregulated MiR‐674‐5p in intestinal epithelial cells is mediated by hypoxia‐inducible factor 1‐alpha (HIF‐1α), which targets XBP‐1, thereby inhibiting the proliferation of intestinal crypt cells [158].

## 4 Diagnosis of Septic Intestinal Injury

### 4.1 Tools for Assessing Intestinal Function

In recent years, the concept of acute gastrointestinal injury (AGI) has been established as a significant component of MODS. AGI is defined as an acute, pathological impairment of gastrointestinal function secondary to critical illness—such as major trauma, burns, extensive surgery or shock—primarily entailing disruption of the mucosal barrier, malabsorption and dysmotility. Widely used clinical scoring systems, such as the gastrointestinal dysfunction (GIF) score, are based on the evaluation of parameters including bowel sounds, diarrhoea, intestinal obstruction, gastric residual volume, gastrointestinal bleeding and intra‐abdominal pressure [36, 159]. A high GIF score within the first 72 h independently predicts mortality in ICU patients, and the AGI grade has also been validated as a strong predictor of 28‐day survival [160]. Nevertheless, these scoring tools remain largely subjective and are constrained by limitations in current monitoring methodologies.

Standardized, clinically validated tools for assessing intestinal epithelial barrier function are lacking. Transepithelial electrical resistance (TER) reflects paracellular resistance via ion passage. The lactulose/mannitol ratio, the most common in vivo permeability test, quantifies mucosa‐to‐serosa flux or blood‐to‐lumen clearance, reflecting paracellular pathway status. This non‐invasive, specific test has been validated in large cohorts for its association with septic gastrointestinal failure [84]. Fluorescent dextran and urinary gluten immunogenic peptide (u‐GIP) probes simplify procedures, broaden monitoring, and enable real‐time assessment of TER‐related barrier function, as validated in animals [84]. However, in vitro TER and permeability measurements are calculated based on serosal surface area (Using chamber aperture) rather than actual mucosal area and also disregard the linear density dependence of paracellular permeability on intestinal length or the crypt‐villus axis [84]. Confocal laser microscopy enables real‐time imaging but is invasive and costly. These methods have not yet entered clinical practice and await validation by large‐scale clinical data.

### 4.2 Quantifiable Laboratory Metrics

Beyond conventional markers like C‐reactive protein (CRP) and platelet count (PLT), a broader spectrum of biomarkers—including fluid‐phase pattern recognition molecules (PRMs), complement components, cytokines, DAMPs, non‐coding RNAs, cell membrane receptors, metabolites, and soluble receptors—is increasingly used to enhance the diagnostic and prognostic assessment of sepsis [161]. Nevertheless, a gold‐standard biomarker specifically indicating intestinal injury during sepsis remains elusive.

Inflammatory and immune alterations serve as direct indicators of the host's immune status and critically influence intestinal barrier integrity. Key mediators of barrier disruption include endotoxins, chemokines and pro‐inflammatory cytokines such as TNF‐α, IL‐1β, IL‐6, IL‐9, and IFN‐γ. In contrast, anti‐inflammatory cytokines like IL‐10 and IL‐22 help maintain intestinal homeostasis and barrier function. Additional circulating markers, including IL‐18, IL‐21, CCL‐3, and tissue inhibitor of metalloproteinase 2 (TIMP‐2), also reflect the state of intestinal homeostasis [13]. However, their utility is often limited by systemic influences, an inability to detect early inflammatory phases, and insufficient gastrointestinal specificity [162]. As highlighted in preceding sections, key signalling pathways involved in septic intestinal injury—such as NF‐κB, cGAS–STING, S100A8/A9, and the itaconate pathway—represent promising candidate biomarkers. Future basic and clinical studies should focus on validating their specificity and translational potential for diagnosing and staging gut‐derived sepsis.

With the progressive exploration of the gastrointestinal barrier's role and metabolic processes, many factors or metabolites derived from the commensal microbiota have emerged as serological markers for gastrointestinal functional impairment [159]. Citrulline and intestinal fatty acid‐binding protein (I‐FABP), both produced specifically by enterocytes, serve as biomarkers of enterocyte mass and mucosal integrity, with circulating levels that reflect intestinal permeability and thereby predict gastrointestinal failure risk in critically ill patients [142, 160]. Diamine oxidase (DAO), an enzyme abundant in intestinal mucosal villi, enters the circulation upon barrier damage and correlates with intestinal bacterial load, though its levels are also modulated by hepatic function via the gut–liver axis [159]. Additional candidate markers include faecal calprotectin (an indicator of mucosal inflammation), trefoil factor (TFF) secreted by goblet cells, and heparanase (involved in glycocalyx degradation)—each showing potential for assessing gastrointestinal injury in sepsis, yet their clinical applicability and mechanistic relevance require further validation [159].

Intestinal hormones such as fibroblast growth factor 15/19 (FGF15/19) and glucagon‐like peptide‐1 (GLP‐1) are key mediators of gut–liver crosstalk. These hormones are closely linked to bile acids—microbiota‐interacting metabolites with emerging roles in enhancing barrier function and limiting bacterial translocation. Circulating levels of FGF15/19 and GLP‐1 thus reflect the functional state of enterohepatic bile acid metabolism [13]. IL‐9 is produced by naive T cells orchestrated by a combination of IL‐4 and TGF‐β, participates in inflammation development via JAK–STAT signalling, reflects CD4^+^ T cell and D‐lactate levels, and is also a potential indicator for predicting 28‐day mortality [163]. Simultaneous measurement of bacterial translocation biomarkers (Lipopolysaccharide‐binding protein (LBP), soluble CD14 subtype (sCD14‐ST)) and intestinal injury markers (I‐FABP, zonulin, regenerating islet‐derived protein 3 alpha (REG3α)) facilitates early prediction and severity stratification of gut‐derived sepsis and predicts liver and cardiovascular injury [164, 165].

### 4.3 Multi‐Omics Differential Characteristics

Faecal microbiome analysis offers a non‐invasive, easily accessible and information‐rich approach for diagnosing intestinal injury in sepsis. During sepsis, gut microbiota show reduced diversity and abundance, an increased Firmicutes/Bacteroidetes ratio, and decreased Proteobacteria/Actinobacteria [8]. Fungal *Candida albicans* decreases, but its metabolite phenylpyruvic acid (PPA) binds sirtuin 2 (SIRT2), increases ROS, enhances macrophage killing, and improves sepsis severity [166]. Metabolomics reveals disturbed amino acid metabolism, with altered tryptophan correlating with disease severity [167]. Peptidomics identifies differentially expressed peptides involved in antigen presentation and MAPK signalling, including apoptosis‐related candidates [22]. Metaproteomics builds functional networks from peptide abundance correlations [168].

Neyton et al. developed a faecal metagenome‐based metabolic dysregulation score (MDS) incorporating SCFAs and aromatic amino acid derivatives; MDS independently predicts 30‐day mortality [169]. Neonatal faecal Shannon diversity and *Enterococcus* abundance correlate with leukocytes, lactate, hospital stay and 28‐day mortality [170]. Luo et al. validated a multimodal strategy combining *Bacteroides salyersiae* abundance, NK cell proportion and CRP, significantly improving prognostic AUC [171].

Faecal metabolomic markers—cytokines, adhesion molecules, H_2_S, substance P, amino acids—predict gut‐target organ axis abnormalities. SCFAs, uremic toxins, bile acids, TMAO mediate cross‐organ immune dysregulation. In aged gut, *Klebsiella aerogenes* increases histamine (HA), causing barrier dysfunction and sepsis susceptibility via HA‐Nlrp6‐LC3 [172].

Electronic nose/GC‐based faecal VOC profiling screens LOS risk in preterm infants, with best prediction for Gram‐negative pathogens [173]. Low‐coverage nanopore sequencing of plasma cfDNA detects microbial translocation and gut barrier injury, enabling non‐invasive concurrent assessment [174]. AI‐driven multi‐omics integration (genomics, transcriptomics, proteomics, metabolomics) could monitor immune status in gut‐derived sepsis, but lacks validation [175].

Improving intestinal function assessment requires (1) establishment of a gold standard for barrier testing, broader application of non‐invasive permeability imaging, and enhanced clinical feasibility of disaccharide probes and (2) promotion of combined faecal omics and serum biomarker panels targeting microbial translocation, barrier dysfunction, and immune phenotypes. Multidimensional data integration will facilitate a multimodal system for accurate sepsis prediction and stratification, pending clinical validation.

Table 1 outlines the monitoring modalities for intestinal injury in sepsis, with a classification of their associated potential biomarkers.

**TABLE 1:** Diagnostic tools and biomarkers for sepsis‐induced intestinal injury.

Category	Biomarker(s)	Specimen/Sample type	Sensitivity	Specificity	Main application scenarios	Advantages	Limitations	References
Scoring scale	First 3D GIF Rating	Clinical signs (bowel sounds, intra‐abdominal pressure, GI bleeding, etc.)	Moderate	Low	Assessment of GI dysfunction and mortality risk stratification in ICU patients	Simple, equipment‐free	Subjective, contraindicated in comatose patients	[36, 160]
	Acute Gastrointestinal Injury (AGI) Score	Clinical signs (gastric residual volume, intra‐abdominal pressure, etc.)	Moderate	Low	Prediction of 28‐day mortality	Simple, equipment‐free	Relies on clinical manifestations, subjective and stochastic	[160]
Barrier function assessment	Transepithelial Electrical Resistance (TER)	Isolated intestinal tissue	High	High	Direct measurement of intestinal epithelial paracellular resistance	Directly reflects mucosal integrity	Invasive, not applicable in routine clinical practice	[84]
	Permeability studies (mannitol/fluorescent dextran, u‐GIP)	Urine (after oral probe administration)	Moderate	Moderate	Assessment of paracellular pathway permeability	Differentiate permeability changes	Absence of defined standards and restricted application	[84]
Inflammatory/immune markers	Pro‐inflammatory factors (TNF‐α, IL‐1β, IL‐6, etc.)	Blood	Moderate	Low	Adjunct assessment of systemic inflammatory status	Mature detection, correlates with systemic inflammation	Lacks intestinal specificity, insufficient early sensitivity	[13, 162]
	Anti‐inflammatory factors (IL‐10, IL‐22, etc.)	Blood	Moderate	Low	Reflects intestinal homeostasis and barrier integrity	Negatively correlated with inflammation	Subject to multiple confounders	[13, 162]
Intestinal‐specific markers	Citrulline	Blood	High	High	Assessment of enterocyte function, prediction of GI failure	Highly specific, clearly associated with GI failure risk	Requires renal function assessment	[142, 160]
	I‐FABP, Zonulin, REG3α	Blood	High	Moderately high	Early detection of intestinal mucosal injury	Early release, short half‐life, reflects acute injury	Affected by extent of intestinal ischemia; possible miss of mild injury	[142, 160, 164, 165]
	DAO	Blood	Moderate	Moderate	Assessment of intestinal barrier integrity	Stable enzymatic activity, correlates with bacterial abundance	Affected by gut‐liver axis; bias in liver dysfunction	[159]
	S100A8/A9	Faeces, blood	Moderately high	Moderately high	Assessment of local intestinal inflammation	Faecal sample better reflects local gut environment	Lower specificity in serum; requires faecal sampling	[159]
	FGF15/19, GLP‐1, bile acids	Blood	Moderate	Moderate	Reflects gut‐liver communication and bile acid metabolism	Therapeutic potential	Limited clinical validation	[13]
	IL‐9	Blood	Moderate	Moderate	Prediction of 28‐day mortality	Correlates with CD4^+^ T cells and D‐lactate	Mechanism not fully elucidated, limited validation	[163]
Microbial translocation	LBP, sCD14‐ST	Blood	High	Moderately high	Early recognition of gut‐derived sepsis and severity stratification	Readily detectable and rapid	Of uncertain clinical value	[164, 165]
Microbiome and multi‐omics	Bacterial communities (Firmicutes/Bacteroidetes, etc.)	Faeces	Moderately high	Moderately high	Reflects gut dysbiosis in sepsis	Non‐invasive, rich information	Relies on metagenomics, technically challenging	[8]
	Fungal community ( *Candida albicans* ) and metabolite PPA	Faeces, blood (metabolites)	Unknown	Unknown	Potential therapeutic target	Novel mechanism (SIRT2‐ROS)	Emerging field, unvalidated	[166]
	Tryptophan metabolic dysregulation	Faeces, blood	Moderate	Moderate	Correlates with sepsis severity	Reflects systemic metabolic phenotype	Lack of standardization in metabolomics	[167]
	Differentially expressed polypeptides/peptide abundance network	Intestinal tissue, faeces	Low	Low	Screening for potential biomarkers, constructing functional networks	Can discover novel biomarkers	Technically complex, mainly for research	[22, 168]
	Metabolic Dysfunction Score (MDS)	Faeces	Moderately high	Moderately high	Independent prediction of 30‐day mortality	Includes actionable factors (short‐chain fatty acids, etc.)	Requires metagenomic sequencing, limited clinical rollout	[169]
	Enterococcus abundance	Faeces	Moderate	Moderate	Neonatal sepsis warning, prediction of 28‐day mortality	Candidate marker, relatively accessible	Mostly in neonates, generalizability to be verified	[170]
	*Bacteroides salyersiae* abundance + NK cell proportion + CRP	Faeces + blood	High	High	Sepsis diagnosis and prognosis	Multi‐modal integration, significantly improved AUC	Requires flow cytometry and metagenomics, high cost	[171]
	Histamine (HA)‐mediated pathway ( *K. aerogenes* ‐HA‐Nlrp6‐LC3)	Faeces, blood	Unknown	Unknown	Gut barrier dysfunction and sepsis susceptibility in aged hosts	Mechanistically clear, potential for targeted intervention	Needs clinical validation	[172]
Emerging non‐invasive technologies	Faecal VOC analysis (e‐nose/gas chromatography)	Faeces	Moderately high	Moderate	Risk screening for late‐onset sepsis in preterm infants	Non‐invasive, rapid, best predictive value for Gram‐negative pathogens	Mainly in neonates, generalizability to be verified	[173]
	Plasma cfDNA nanopore sequencing	Blood	Moderate (57%)	Moderate (73%)	Simultaneous assessment of multi‐organ damage and pathogens	Single assay provides multi‐dimensional information	Sensitivity needs improvement, high cost	[174]

## 5 Potential Therapeutic Strategies for Sepsis‐Induced Intestinal Injury

### 5.1 Repair of the Intestinal Barrier and Special Pharmacokinetics of Gastrointestinal Drugs

Preserving the intestinal mucosa is a critical measure for mitigating intestinal injury. Intestinal peptides can pharmacologically modulate the intestinal mucosa. Cathelicidin‐BF, an antimicrobial peptide purified from *Bungarus fasciatus venom*, mediates its protective role by downregulating NF‐κB to prevent ZO‐1/occludin redistribution, suppressing macrophage‐derived NF‐κB/TNF‐α activation, and reducing intestinal epithelial cell apoptosis [176]. FGF19, an enterokine that inhibits bile acid synthesis and reverses lipogenesis, protects against LPS‐induced injury in the intestine and liver. By improving serum linoleic and γ‐linolenic acid levels and reducing oxidative stress, this effect positions FGF19 as a promising therapeutic target for sepsis [177]. FXR agonists can promote microbiota reconstruction and restore epithelial barrier function. The FXR agonist obeticholic acid (OCA) targets HIF1α to modulate the maturation of MDSCs, exhibiting a preventive effect against sepsis [178]. It also targets the FXR‐FGF15 signalling axis to improve bile acid metabolism and alleviate gut‐liver axis disruption [98].

The gastrointestinal tract possesses unique digestive and motor functions, imposing specific requirements for drug stability and solubility when targeting this system. To enable targeted intervention within the intestinal lumen, hydrogel technology utilizing cellulose cross‐linked with citrate can mimic natural dietary fibres. Furthermore, insoluble polymeric drugs and carbon nanoparticles within the intestine can selectively and with high affinity bind to bacteria/toxins and inorganic ions, enabling adsorption and clearance. Nonselective beta‐blockers can shorten intestinal transit time and reduce permeability, thereby decreasing the bacterial load [98]. Endoscopic procedures such as duodenal mucosal resurfacing or ablation limit nutrient‐mucosa contact, thereby modulating aberrant signalling in insulin‐sensitive tissues and offering a mechanical approach to metabolic intervention [98].

### 5.2 Antioxidant and Anti‐Inflammatory Therapy

Oxidative stress and inflammation, as critical initiating events in intestinal injury during sepsis, render targeted antioxidant and anti‐inflammatory therapies a potential strategic approach for early intervention to halt sepsis progression and mitigate organ damage. Possessing enzyme‐like activity that mimics SOD, single‐atom nanozymes (SAzymes), exemplified by a copper‐containing variant, catalytically eliminate superoxide radicals with high efficiency. This action disrupts the associated radical chain reactions and attenuates the inflammatory cascade during the early phase of sepsis [179]. The Nrf2‐Keap1 pathway, a central regulator of oxidative stress response, serves as a primary target for numerous antioxidant herbal extracts [55, 115]. Kaixin Ping et al. found that Gyp XLIX significantly inhibits ROS formation while modulating the Nrf2‐Keap1 pathway. Furthermore, emodin and liensinine reduce oxidative stress by activating the Nrf2 pathway, leading to upregulated antioxidant enzymes (HO‐1, SOD, GSH‐Px, CAT) and decreased lipid peroxidation (MDA), thereby enhancing overall antioxidant capacity (T‐AOC) [20, 55].

Mitochondrial integrity is also crucial; deficiency in the uncoupling protein UCP2 exacerbates oxidative stress and inflammation, marked by elevated ROS and MDA, decreased SOD and GSH‐Px activity, pro‐inflammatory cytokine release, and reduced IL‐10 [117]. The E3 ubiquitin ligase CHIP plays a key role in modulating oxidative stress. Geranylacetone (GGA), an anti‐ulcer agent, upregulates HSP70 to promote the CHIP‐mediated ubiquitination and degradation of KPNA2, thereby inhibiting NF‐κB nuclear translocation and mitigating oxidative stress and inflammation [141]. Similarly, the novel compound YL‐109 upregulates CHIP expression, inhibits the ERK/AP‐1 and NF‐κB/NLRP3 pathways, reduces pyroptosis and mitigates intestinal injury [142]. Combination therapy with these agents may yield enhanced efficacy. Faecal metabolomics identified the lipid metabolite PE(0:0/14:0) as an upregulator of tight junction protein expression, which functions by activating the AhR/cytochrome P450 1A1 (CYP1A1) pathway [180].

Postbiotics—defined as functional metabolic byproducts of gut microbiota including SCFAs, secondary bile acids, proteins, polysaccharides, and vitamins—act as key metabolic modulators [98]. Notably, one of the SCFA NaB inhibits NF‐κB p65 nuclear translocation, upregulates IκB‐α expression, and suppresses NF‐κB activity [121]. Exogenous supplementation with enteric glial cell‐derived S‐nitrosoglutathione (GSNO) reduces pro‐inflammatory cytokine levels, inhibits LPS‐induced upregulation of intestinal myosin light chain kinase expression and NF‐κB p65 levels and decreases permeability [181]. Modulating intestinal arginine and nitric oxide metabolism may mitigate age‐related intestinal barrier impairment and inflammatory responses [182].

Sepsis patients display significant upregulation of programmed death‐1/programmed death ligand‐1 (PD‐1/PD‐L1) on peripheral blood leukocytes, contributing to immune dysfunction. Granulocyte‐macrophage colony‐stimulating factor (GM‐CSF) can counteract this immunosuppression, while anti‐PD‐L1 antibody therapy reduces immune cell apoptosis, improves leukocyte function and restores tight junction integrity, thereby alleviating TNF‐α/IFN‐γ‐induced inflammatory injury [183]. Furthermore, inhalation of 2% hydrogen gas exerts antioxidant, anti‐inflammatory and anti‐apoptotic effects, improves thyroid and nitrogen metabolism and ameliorates intestinal injury [22]. Human recombinant B‐type natriuretic peptide (rhBNP) mediates its protective effect against LPS‐induced ALI through inhibition of IκB phosphorylation and downregulation of pro‐inflammatory cytokines such as TNF‐α and IL‐6 [184]. Limonene, a monocyclic monoterpene hydrocarbon, primarily targets the MyD88‐dependent pathway in the inflammatory cascade, reducing the production of inflammatory cytokines by inhibiting the TLR4/NF‐κB/AP‐1 pathway [124]. MicroRNAs (miRNAs) are short (~22 nt) endogenous non‐coding RNA molecules that play critical roles in post‐transcriptional gene regulation [158]. Omega‐3 fatty acids, as nutraceuticals derived from TCM, regulate the miR‐1‐3p/Notch3/Smad axis, inhibit Smad signalling activation and alleviate inflammation, oxidative stress and apoptosis [185].

### 5.3 Immunomodulatory Therapy

Immunoparalysis represents a pivotal factor in multiorgan injury during the late phase of sepsis. Notably, conditions such as macrophage activation‐like syndrome (MALS) and immunoparalysis are now delineated as stratification markers for individualized adjunctive immunotherapy in septic patients. Restoring immune homeostasis constitutes a critical therapeutic strategy for immunomodulation in sepsis‐associated intestinal injury, as emphasized in the works of scholars including Konstantinos Leventogiannis [186, 187]. In a study by Xuancheng Du et al., immunomodulatory nanozymes designated as PdIr@OMVs function as immunostimulatory carriers. This construct enables targeted delivery to the gut, where it enhances localized peroxidase‐like catalytic activity, potentiating the phagolysosomal bactericidal capacity of impaired macrophages, facilitating the eradication of intracellular MDR bacteria. Subsequent antigen release promotes DC activation and antigen presentation, thereby re‐engaging innate immunity under immunoparalytic conditions, attenuating bacterial burden, and reestablishing immune homeostasis [188]. Apolipoprotein A1 (apoA1)—IL4‐conjugated lipid nanoparticles specifically target myeloid immune cells and reverse immunoparalysis in sepsis through trained immunity [189]. In murine sepsis, mitochondrial transplantation improved mitochondrial dysfunction and reduced apoptosis, thereby mitigating excessive inflammation and immune paralysis while enhancing survival rates and bacterial clearance [190].

Macrophages, as the predominant immune cells in the intestinal lamina propria, undergo M1/M2 polarization, which represents a crucial target for modulating immune homeostasis. Fisetin, a berry‐derived flavonoid, suppresses pro‐inflammatory cytokine expression in macrophages via inhibition of the TAK1/p38 MAPK/MK2 axis [129]. MiRNAs critically regulate macrophage polarization, with specific members driving distinct phenotypes: miR‐23a (via TLR/IFN suppression) and miR‐27‐3p (via PPAR‐γ targeting) promote M2 polarization, whereas miR‐211 (through STAT1/3/SOCS1) and miR‐3061 (by inhibiting Snail) modulate M1 polarization [131]. Other miRNAs, including miR‐222, miR‐322/503, miR‐21/155, miR‐195, miR‐122b and miR‐29b, can regulate intestinal epithelial cell proliferation, apoptosis and intercellular interactions. HIF‐α induces MiR‐674‐5p, which targets XBP‐1, thereby inhibiting intestinal crypt cell proliferation and exacerbating gut injury [158]. Novel nucleic acid therapeutics such as tetrahedral framework nucleic acids (tFNAs)—programmable nanostructures assembled from DNA strands—can activate the Mertk/STAT1/SOCS pathway to direct macrophage polarization towards the M2 phenotype [130]. Furthermore, macrophage‐derived extracellular vesicle mimetics (EVM) prepared by membrane extrusion technology are rich in functional proteins from M1/M2 macrophages. They can promote fibroblast fibrotic phenotypes, enhance angiogenesis and suppress excessive M1 polarization, which has been validated for improving the survival rate of bioengineered tissues. These mimetics, functioning as a targeted drug delivery platform, offer considerable potential for the targeted management of sepsis‐induced intestinal injury, meriting additional preclinical and clinical study [191].

Shifts in immune cell populations also reflect immunological status. Berberine augments Treg‐mediated immunosuppression in mesenteric lymph nodes by promoting Treg expansion and upregulating FoxP3/CTLA‐4 [9]. Inhibiting lymphocyte apoptosis represents another immunotherapeutic strategy: IL‐7 expands CD3^+^ T cell numbers and reverses immunometabolic dysfunction via mTOR signalling; β‐blockers modulate lymphocyte homeostasis; and miRNAs like miR‐223 or immune checkpoint inhibitors (anti‐PD‐1/PD‐L1) reduce apoptosis and counter immunosenescence in sepsis [76].

Prophylactic vaccination represents a promising strategy for modulating intestinal barrier function and gut microbial metabolism, though its progress has been constrained by limitations in oral bioavailability and targeted delivery. Recent innovations in oral nanomedicine have improved the selective transport of therapeutic agents—such as antigens, immunomodulators, barrier enhancers, and microbiota‐modulating compounds—across the gastrointestinal tract. These platforms overcome physiological and biochemical barriers, enable site‐specific drug delivery, and harness immune activation or tolerance induction to achieve effective oral vaccination. The ability of nanomedicine to strengthen the intestinal barrier and modulate gut microbiota/metabolites shows significant potential for applications spanning oral vaccines, immune tolerance, inflammatory bowel disease and systemic conditions like cancer [12, 192].

### 5.4 Regulation of PCD in IECs

Targeted inhibitors of programmed cell death also demonstrate potential in alleviating intestinal injury. Research by YiKun Chen et al. revealed that emodin modulates the JAK/STAT signalling pathway to regulate the expression of Bcl‐2 and Bax, thereby reducing intestinal epithelial cell apoptosis and decreasing levels of procalcitonin (PCT), IL‐6 and TNF‐α [146].

Liensinine, extracted from the embryo of lotus seeds, ameliorates intestinal morphological damage, suppresses inflammatory responses and mitigates oxidative stress via the Nrf2‐Keap1 pathway. It functions primarily through the PI3K/AKT/mTOR pathway to alleviate LPS‐induced autophagy impairment. Additionally, it attenuates apoptosis and pyroptosis, thereby reducing intestinal barrier disruption [115]. Pharmacological inhibition of necroptosis also shows protective effects. Necrosulfonamide blocks MLKL pore formation and subsequent STING signalling, inhibiting interferon mRNA induction. Similarly, necrostatin‐1 has been demonstrated to suppress necroptosis, enhance barrier function and reduce pro‐inflammatory cytokine levels and macrophage infiltration in intestinal injury models [145, 193]. Innovative delivery systems further enhance therapeutic potential. Yan et al. developed an intestinal deciphering engineered capsule that coats macrophages onto polymer‐based organic nanoparticles encapsulating olaparib. Upon effective delivery to the intestine, the released nanoparticles utilize macrophage membrane receptors to effectively neutralize pro‐inflammatory cytokines. Simultaneously, olaparib inhibits parthanatos in intestinal epithelial cells by suppressing poly (ADP‐ribose) polymerase 1 activation, thereby effectively reducing bacterial translocation and mitigating the onset of sepsis [194].

### 5.5 Microbiome Abundance Modulation

Probiotics, particularly strains such as *Lactobacillus rhamnosus* , have demonstrated the capacity to mitigate pathogen invasiveness and transcytosis—exemplified by pathogenic *Escherichia coli* —enhance tight junction protein expression, maintain barrier integrity, and reduce bacterial translocation. They further modulate intestinal T cell responses, attenuate inflammatory factor secretion by epithelial cells, and SIgA production, thereby limiting bacterial adhesion and strengthening mucosal immunity. However, the certainty of probiotic efficacy in sepsis remains low to moderate, with no consistent evidence for attenuation of apoptosis or tissue injury [195, 196].

Specific probiotic combinations, including *Bifidobacterium bifidum* E3 and *Bifidobacterium longum subsp. Infantis* E4, ameliorate LPS‐induced intestinal injury by suppressing TLR4/NF‐κB and MAPK signalling. These strains reinforce intestinal barrier function and tight junctions, improve CD4^+^ T cell proportions and CD4^+^/CD8^+^ ratios, inhibit phosphorylation of p38, c‐Jun N‐terminal kinase, and ERK1/2 and reduce pathogenic bacterial abundance [197]. Synthetic live bacteria, also referred to as engineered probiotics, are designed to selectively convert toxic metabolites into nontoxic forms [98]. Metabolomic analyses of gut microbiota have identified significant enrichment in valine, leucine and isoleucine biosynthesis pathways during injury, with a marked depletion in beneficial metabolites such as L‐valine. Exogenous L‐valine administration upregulates tight junction proteins including ZO‐1 and alleviates intestinal damage [198].

The misuse of broad‐spectrum antibiotics is a key factor driving intestinal bacterial translocation. Selecting antibiotics based on specific antimicrobial susceptibility represents a rational strategy to limit this effect. For instance, rifaximin—a non‐absorbable antibiotic targeting Gram‐negative bacteria—reduces endotoxin levels and exerts anti‐inflammatory activity [98]. Lolamicin, a gram‐negative pathogen‐specific antibiotic, selectively eliminates pathogenic bacteria while preserving commensal microbiota by targeting the lipoprotein transport system, thereby achieving dual selective antimicrobial action [199]. Beyond conventional antibiotics, several targeted approaches show promise. Bacteriophages, which specifically infect and lyse pathogenic bacteria, enable precise pathogen eradication without promoting antibiotic resistance [98] The phage cocktail AB‐SA01, for example, has demonstrated favourable safety in patients with severe *Staphylococcus aureus infections* [200]. Faecal microbiota transplantation (FMT) can restore a healthy microbial ecosystem; antibiotic preconditioning has been shown to improve the engraftment efficiency of donor microbiota [201].

Synthetic biology represents a novel therapeutic paradigm for addressing antimicrobial resistance (AMR) by enabling the rational screening and assembly of diverse biological components into engineered, targeted living systems. The strategic engineering of living therapeutics—including phages, microbes and human cells—demonstrates considerable potential as a promising strategy for advanced antimicrobial therapy [202]. Emerging engineered probiotics leverage inter‐organ pathways such as the gut–lung axis for systemic immunomodulation. One such approach uses engineered *E. coli* ΔPESI strains that stably express interleukin‐1 receptor antagonist (IL‐1Ra) via polyethylene glycol–modified outer membrane vesicles, enabled by the SpyCatcher–SpyTag conjugation system. These engineered strains enhance intestinal barrier function, suppress inflammation, modulate macrophage polarization and pyroptosis in the lungs and attenuate systemic inflammatory responses [203].

### 5.6 ISC Transplantation and Tissue Repair

Leveraging the intrinsic proliferative and differentiation potential of ISCs, gut‐on‐a‐chip systems offer dynamic, physiologically relevant platforms to model the intestinal epithelium and its microenvironment. These microengineered devices enable detailed investigation of host–microbiome interactions under controlled conditions [204].

Microvesicles derived from mesenchymal stem cells—particularly membrane‐derived microvesicles (MMVs)—contribute to the restoration of intestinal barrier integrity by improving mitochondrial quality and function. MMVs deliver key regulators such as MFN2 and peroxisome proliferator‐activated receptor gamma coactivator 1‐alpha (PGC‐1α) to intestinal epithelial cells, enhancing mitochondrial fusion and biogenesis. Furthermore, they facilitate the direct transfer of functional mitochondria to epithelial cells, boosting energy metabolism and supporting barrier recovery [205]. MMV also functions as a regulatory factor that targets and enhances immune responses while promoting microbial reconstitution [206]. The combined administration of MMV and azithromycin effectively mitigates infection, modulates inflammatory responses and alleviates organ damage [207]. *Lactobacillus rhamnosus* GG (LGG) promotes ISCs' regeneration and enhances gut barrier integrity by upregulating the expression of Lgr5^+^ and lysozyme [208].

### 5.7 The Role of Integrated TCM Therapy in Sepsis‐Induced Intestinal Injury

While chemically synthesized drugs are widely utilized due to their rapid efficacy and low‐dose advantages, issues such as drug resistance and their inhibitory effects on proton pumps cannot be overlooked. In contrast, TCM‐based approaches targeting the intestinal barrier and microbiota may offer potential strategies for modulating gut health and promoting tissue repair [209]. The ‘Xuanfei Tongfu’ method, rooted in the TCM principle of the exterior‐interior relationship between the lung and large intestine, has been applied in clinical practice. Early intervention with Xuanbai Chengqi Decoction has shown efficacy in reducing intestinal‐derived endotoxemia [9].

Similarly, the ‘Jianpi Hewei’ method promotes tolerance to enteral nutrition and protects the mucosal barrier, which aligns with the traditional principle of reinforcing healthy qi (host immunity) to eliminate pathogenic factors. Numerous TCM formulations and botanical extracts derived from Chinese herbs have demonstrated efficacy in repairing intestinal injury associated with sepsis. Dongze Qiu et al. reported that berberine (BBR), a natural isoquinoline alkaloid, ameliorates intestinal inflammation by enhancing Treg‐mediated immunosuppression via FoxP3/CTLA‐4 upregulation, which rebalances cytokine production towards an anti‐inflammatory state (inhibiting IL‐1β/IL‐6/TNF‐α and promoting IL‐10) [9].

Selenium‐rich peptides from cardamom modulate mitochondrial fusion via MFN2, improving intestinal morphology, disaccharidase activity and tight junction protein expression, while suppressing IL‐6 release [210]. Rhubarb stabilizes vascular endothelial integrity by regulating vascular endothelial cadherin expression and myosin light chain phosphorylation [36]. The dammarane‐type saponin Gyp XLIX (from *Gynostemma pentaphyllum*, used in TCM for chronic bronchitis) mitigates oxidative stress in septic intestinal injury by inhibiting ROS, activating Nrf2‐Keap1 to reduce MDA and elevating antioxidant capacity. It also suppresses the NF‐κB pathway and NLRP3 inflammasome activation, thereby mitigating intestinal inflammation. Additionally, it activates the PI3K‐AKT pathway, inhibiting intestinal cell apoptosis and excessive autophagy, which helps alleviate intestinal damage [55].

Essential oil from *Chimonanthus nitens* Oliv (CEO) reduces mitochondria‐associated endoplasmic reticulum membrane formation and NLRP3 localization, exerting anti‐inflammatory and antioxidant effects that are enhanced by MFN2 knockdown [140]. Corilagin, a polyphenolic tannin, binds to Bip and promotes its ubiquitin‐dependent degradation, inhibiting endoplasmic reticulum stress and alleviating intestinal ischemia–reperfusion injury [211]. Baiwei Baidu Powder increases *Lactobacillus johnsonii* abundance and modulates macrophage anti‐inflammatory activity, mitigating sepsis‐induced liver injury [212].

Studies highlight natural foods as important sources of anti‐inflammatory agents. Zhiling Li et al. showed that naringin, a citrus flavanone, improves intestinal mucosal barrier function and attenuates inflammation by modulating the Ras homologue family member A (RhoA)/Rho‐associated coiled‐coil containing protein kinase (ROCK)/NF‐κB/myosin light chain kinase (MLCK)/myosin light chain (MLC) pathway to elevate tight junction proteins (ZO‐1, claudin‐1) and inhibiting macrophage release of TNF‐α and high mobility group box 1 (HMGB1) [213]. Furthermore, naringin may promote M2 macrophage polarization by targeting the PPARγ/miR‐21/STAT1/6 signalling axis [214]. Quercetin activates the Nrf2/HO‐1 pathway, enhances barrier function, inhibits NLRP3 inflammasome activation and promotes microbial diversity [215].

Acupuncture and moxibustion, collectively termed acupuncture therapy, involve the stimulation of specific acupoints on the body using fine needles or moxa cones to prevent or treat disease. As a cornerstone of TCM, acupuncture is widely employed in clinical practice and has demonstrated efficacy in ameliorating conditions such as gastroparesis, enhancing gastrointestinal motility, reducing intra‐abdominal pressure, and improving gastrointestinal dysfunction. The therapeutic mechanisms may involve the suppression of pro‐inflammatory factor release, promotion of anti‐inflammatory mediators and polyamines, and modulation of macrophage polarization, although the precise molecular pathways require further elucidation [44].

**FIGURE 6:**
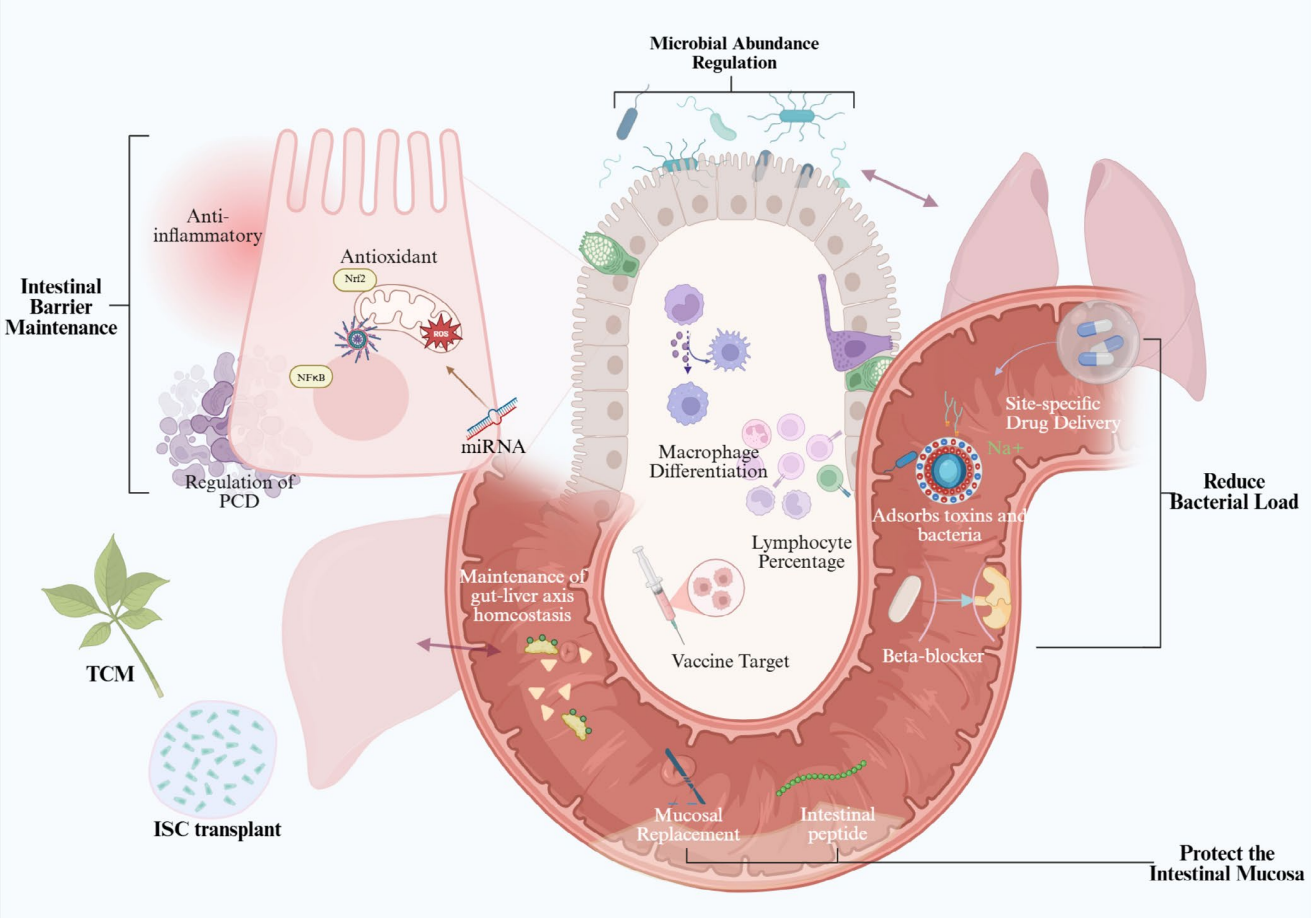
Diagram of potential therapeutic targets for sepsis‐induced intestinal injury. The multi‐layered and complex regulatory network underlying sepsis‐induced intestinal injury offers a broad spectrum of therapeutic targets, with a primary focus on modulating microbial abundance and preserving the intestinal barrier. Barrier maintenance involves direct anti‐inflammatory and antioxidant therapies, alongside the regulation of PCD. Deeper therapeutic strategies include the application of mRNA‐based technologies, promoting maturation of the gut‐liver axis, utilizing TCM, and performing intestinal stem cell transplantation for tissue repair. Targeting the microbiota itself involves microbial replacement (e.g., probiotics, faecal microbiota transplantation) and precise elimination of pathogenic bacteria (e.g., targeted phage therapy) to restore a healthy microbial ecosystem. Furthermore, interventions utilizing gut peptides and β‐blockers—which can reduce bacterial load—combined with site‐specific drug delivery technologies, enable precise protection of the intestinal mucosa and collectively form a multi‐targeted integrated therapeutic network.

Figure 6 synthesizes therapeutic strategies targeting the pathophysiological alterations and molecular drivers of intestinal injury in sepsis, while Table 2 categorizes therapeutic targets and reviews drug development advances for intestinal injury in sepsis, supporting the feasibility of multi‐level optimized management.

**TABLE 2:** Sepsis‐induced intestinal injury: Therapeutic approaches and potential targets.

Category	Therapeutic strategy	Therapeutic modality/procedure	Drug/Agent therapy	Route of administration	Gut‐targeted release	Drug target/signalling pathway	References
Barrier restoration and site‐specific drug kinetics	Intestinal barrier protection and repair	Antimicrobial peptide Therapy	Cathelicidin‐BF (antimicrobial peptide)			Downregulates NF‐κB, prevents redistribution of ZO‐1 and occludin in IECs, inhibits NF‐κB and TNF‐α activation in macrophages, and reduces IEC apoptosis.	[176]
	Metabolic Regulation and Bile Acid Homeostasis	FGF19 (Fibroblast growth factor 19)	FGF19		Gut‐derived	An intestinally‐derived inhibitor of bile acid biosynthesis that improves serum fatty acid (LA, GLA) levels and alleviates oxidative stress.	[177]
		FXR agonist	Obeticholic acid (OCA)		Targeting intestinal FXR	Targets HIF‐1α to regulate myeloid‐derived suppressor cell (MDSC) maturation; acts on the FXR‐FGF15 signalling axis to improve bile acid metabolism and alleviate gut‐liver axis dysregulation.	[98, 178]
	Physical Sequestration and Detoxification	Sorbent technology	Modified cellulose Hydrogels, Insoluble cross‐linked polymers, carbon nanoparticles	Oral	Yes	Targeting HIF‐1α to regulate MDSC maturation; modulating FXR‐FGF15 signalling to improve bile acid metabolism and alleviate gut‐liver axis disruption.	[98, 178]
	Regulation of gut motility and barrier function	Pharmacologic intervention	Non‐selective beta‐blockers	Oral		High‐affinity binding to bacteria/toxins, enabling adsorption and clearance.	[98]
	Reduction of mucosal exposure	Surgical intervention	Duodenal mucosal resurfacing/mucosal ablation	Surgery	Local action	Shortens intestinal transit time, reduces permeability, and decreases bacterial load.	[98]
Antioxidant and anti‐inflammatory therapy	Activate antioxidant pathway	Herbal medicine extract	Gypenoside XLIX (Gyp XLIX), emodin, rhynchophylline			Modulation of the Nrf2‐Keap1 pathway to activate downstream antioxidant enzymes such as HO‐1, SOD, GSH, and CAT, and reduce MDA.	[20, 55, 115]
	Regulation of mitochondria and ubiquitinated proteins	Pharmacotherapeutic intervention	Geranylacetone (GGA)			Upregulation of HSP70 to promote CHIP‐mediated ubiquitination and degradation of KPNA2, thereby inhibiting NF‐κB nuclear translocation	[141]
		Novel compound	YL‐109			Upregulation of CHIP expression to inhibit the ERK/AP‐1 axis and the NF‐κB/NLRP3 inflammasome pathway.	[142]
	Modulation of lipid metabolism and receptors	Metabolite therapy	Lipid metabolite PE(14:0/0:0)			Upregulation of tight junction protein expression via the AHR/CYP1A1 pathway.	[180]
	Postbiotic therapy	Short‐chain fatty acids (SCFAs)	Sodium butyrate (NaB)			Inhibition of NF‐κB p65 nuclear translocation and upregulation of IκBα expression.	[121]
		Gas molecular therapy	Inhalation of 2% hydrogen (H_2_)	Inhale		Antioxidant, anti‐inflammatory, and anti‐apoptotic effects, with improvement in thyroid hormone and nitrogen metabolism signalling.	[22]
	Immunomodulation of immune checkpoints and cytokines	Antibody/protein therapy	PD‐L1 antibody, granulocyte‐macrophage colony‐stimulating factor (GM‐CSF)			Reduction of immune cell apoptosis, improvement in cellular function, and restoration of tight junctions.	[183]
			Human recombinant brain natriuretic peptide (rhBNP)	Vein		Inhibition of IκB phosphorylation and downregulation of TNF‐α and IL‐6.	[183]
	Targeting inflammatory signalling pathways	Natural compound	Limonene			Inhibition of the TLR4/NF‐κB/AP‐1 pathway.	[124]
	miRNA and nutrient regulation	Dietary nutrient	Omega‐3 fatty acid(s)	Oral		Modulation of the miR‐1‐3p/Notch3/Smad axis to inhibit Smad signalling activation.	[185]
Immunomodulatory therapy	Regulation of macrophage polarization	Dietary flavonoid	Fisetin	Oral		Inhibition of the TAK1/p38 MAPK/MK2 signalling pathway.	[129]
		Nucleic acid nanoparticle	Tetrahedral framework nucleic acid (tFNAs)	Nanodrug	Programmability	Targeted activation of the Mertk/STAT1/SOCS signalling pathway to regulate M1/M2 polarization.	[130]
	Regulation of lymphocyte function	Botanical drug extract	Berberine (BBR)	Oral	For intestinal diseases	Increase in Treg cell population, upregulation of FoxP3 and CTLA‐4 expression, and inhibition of pro‐inflammatory factors.	[9]
		Cytokine/antibody	IL‐7, β‐adrenergic blocker, anti‐PD‐1/PD‐L1 antibodies			Reversal of lymphopenia, correction of immunometabolic dysregulation, and reduction of lymphocyte apoptosis.	[76]
	Oral vaccines and nanomedicine	Nanodrug delivery	Oral Nanomedicine (multiple carriers)	Oral	Yes	Selective delivery of antigens and immunomodulators to the gut to activate immunity and modulate the microbiota.	[12, 192]
Regulation of intestinal epithelial cell programmed cell death	Inhibition of apoptosis	Botanical drug extract	Emodin			Modulation of the JAK/STAT pathway to regulate Bcl‐2/Bax, reducing intestinal epithelial cell (IEC) apoptosis.	[146]
	Inhibition of autophagy and pyroptosis	Botanical drug extract	Liensinine			Acting on the PI3K/Akt/mTOR pathway to reverse autophagy inhibition and alleviate apoptosis and pyroptosis.	[115]
	Inhibition of necroptosis	Small molecule inhibitor	Necrosulfonamide (NSA), Nec‐1			NSA disrupts MLKL pore formation and inhibits STING signalling; Nec‐1 suppresses necroptosis.	[145, 193]
	Inhibition of parthanatos	Nanodrug	cp‐OLA@MΦ NPs (Olaparib nanoparticles)	Oral	Engineered targeting	Neutralization of pro‐inflammatory factors, inhibition of PARP1 activation, and suppression of intestinal epithelial parthanatos.	[194]
Modulation of microbiota abundance	Probiotic supplementation	Probiotic	*Lactobacillus rhamnosus* , Bifidobacterium	Oral	Yes	Increase in tight junction proteins, modulation of T cells, elevation of SIgA, and inhibition of the TLR4/NF‐κB/MAPK pathway.	[195, 196]
	Supplementation of beneficial metabolites	Amino acid supplementation	L‐valine			Increase in the expression of barrier proteins such as ZO‐1.	[198]
	Precise antibacterial	Phage therapy	Phage cocktail AB‐SA01		Specificity	Precise lysis of *Staphylococcus aureus* without inducing antibiotic resistance.	[98, 200]
	Microbiota reconstruction	Faecal microbiota transplantation (FMT)	Healthy donor microbiota	Transplantation	Yes	Restoration of physiological microbiota colonization.	[201]
	Engineered bacterial therapy	Engineered probiotic	ΔPESI *E. coli* (producing IL‐1Ra)	Oral	PEGylated colonization	Enhancement of barrier function, inhibition of inflammation, modulation of pulmonary macrophage polarization, and alleviation of systemic inflammation.	[203]
Intestinal stem cell transplantation and tissue repair	Promotion of tissue repair and regeneration	Stem cell therapy	Mesenchymal stem cell microvesicles (MMVs)		Nanovesicle delivery	Delivery of MFN2 and PGC‐1α to promote mitochondrial fusion and biogenesis, thereby improving energy metabolism.	[205]
	Experimental model	Gut‐on‐a‐chip	Platform technology			Providing a dynamic and physiologically relevant model for studying microbiome‐host interactions.	[206]
Integrated traditional Chinese medicine therapy	Traditional Chinese medicine formulas and treatment principles	Lung‐diffusing and fu‐organ‐unblocking method	Xuanbai Chengqi Tang	Oral		Reduction of gut‐derived endotoxemia based on the theory of ‘exterior‐interior relationship between the lung and the large intestine’.	[9]
		Spleen‐fortifying and stomach‐harmonizing method		Oral/enteral nutrition		Protection of the mucosal barrier and ‘fortifying the healthy qi to eliminate pathogenic factors’.	[9]
	Traditional Chinese medicine and natural products	Multiple extracts	Berberine (BBR), selenium‐enriched peptides, gypenoside XLIX, and others	Oral	Multiple natural ingredients	Comprehensive effects: anti‐inflammatory, antioxidant, immunomodulatory, barrier‐protective, etc.	[9, 55, 209, 210, 211, 212, 213, 214, 215]
	Physical therapy	Acupuncture	Acupuncture needles/moxa	Cutaneous stimulation	Meridian	Enhancement of gastrointestinal motility, reduction of intra‐abdominal pressure, immunomodulation, and downregulation of pro‐inflammatory factors.	[44]

## 6 Conclusions and Perspectives

The gastrointestinal tract represents a critical—yet historically understudied—target organ in sepsis, particularly when compared to the heart, lungs, kidneys and liver. Nonetheless, its central role in the pathogenesis and progression of sepsis is increasingly recognized. Early monitoring and intervention for intestinal injury have emerged as pivotal strategies to improve patient outcomes and prevent MODS. This review systematically outlines the core pathophysiological mechanisms underlying septic intestinal injury, which include ischemia–reperfusion injury resulting from systemic blood flow redistribution, oxidative stress and disruption of the local microenvironment. These initiating events trigger dysregulated programmed cell death in intestinal epithelial cells, immune cell dysfunction and compositional shifts within the lamina propria, and ultimately a comprehensive failure of the intestinal barrier. This failure is manifested as endothelial dysfunction, downregulation of tight junction proteins, impaired secretion of MUC, IAP and AMPs, and altered macrophage polarization. Together, these alterations increase intestinal permeability and promote bacterial translocation, driving the onset of ‘gut‐derived sepsis’.

Furthermore, the gut functions as a key ‘driver’ of sepsis progression, exacerbating remote organ injury through bidirectional crosstalk via the gut–liver, gut–brain, and gut–lung axes. These interactions provide new pathophysiological insights into sepsis‐associated encephalopathy, acute liver injury and ALI. The processes described above are governed by complex molecular networks marked by substantial cross‐talk and synergy between signalling pathways. Key pathways and molecular targets highlighted in this review not only represent promising biomarkers but have also received preliminary validation in experimental models, thereby laying a groundwork for clinical translation and illuminating new directions for therapeutic innovation.

Given that microbial translocation and immune dysregulation subsequent to intestinal injury are pivotal hubs for systemic inflammatory response and organ damage, developing efficient monitoring tools and targeted interventions is crucial for assessing sepsis severity and improving prognosis. Current first‐line clinical monitoring indicators, such as inflammatory parameters and humoral/cellular immune markers, lack sufficient specificity and sensitivity for intestinal injury. A future multimodal assessment paradigm—integrating microbiome profiling, intestinal function evaluation, and immune phenotyping—is warranted to establish diagnostic criteria and subtyping for gut‐derived sepsis. Leveraging non‐invasive technologies and high‐specificity/high‐sensitivity biomarkers, this approach will underpin prognostic stratification and precision intervention.

Notably, antibiotic resistance in ICU patients exacerbates gut microbiota dysbiosis and injury, and current Western medical therapies remain limited in effectively restoring intestinal barrier function. Collectively, future therapeutic strategies should focus on the following directions: First, developing targeted agents against key pathways in septic intestinal injury and advancing their clinical validation, while exploring combination therapies with TCM and its active constituents to enhance efficacy. Second, optimizing drug delivery systems (e.g., for small molecule inhibitors, miRNAs, tetrahedral framework nucleic acids) to ensure effective local intestinal drug concentrations and systematically evaluating their biochemical stability and biosafety. Third, utilizing metabolomic and peptidomic analyses to identify differential alterations in the gut microbiota, thereby screening key molecular mechanisms and reducing microbial burden, ultimately enabling precise, personalized treatment. Through these concerted efforts, we can significantly narrow the translational gap in the management of septic intestinal injury.

## Author Contributions

**Yichen Bao:** drafting and writing of manuscript, drawing of figures. **Lin Qi:** drawing of tables and figures. **Guijun Zou:** drafting and writing of manuscript. **Xingpeng Yang:** bibliographic retrieval. **Yizhao Ma:** bibliographic retrieval. **Zhifu Li:** organization of references. **Xiaohui Du:** conception and design of the work, revision and final approval of the version to be published. **Pengyue Zhao:** conception and design of the work, revision of manuscript.

## Funding

This work was supported by the National Natural Science Foundation of China (Nos. 82372158, 82502590).

## Consent

All of the authors are aware of and agree to the content of the paper and to being listed as co‐authors.

## Conflicts of Interest

The authors declare no conflicts of interest.

## Data Availability

Data sharing not applicable to this article as no datasets were generated or analysed during the current study.
